# Genetic dissection of grain water content and dehydration rate related to mechanical harvest in maize

**DOI:** 10.1186/s12870-020-2302-0

**Published:** 2020-03-17

**Authors:** Jianju Liu, Hui Yu, Yuanliang Liu, Suining Deng, Qingcai Liu, Baoshen Liu, Mingliang Xu

**Affiliations:** 1 State Key Laboratory of Plant Physiology and Biochemistry/College of Agronomy and Biotechnology/National Maize Improvement Center/Center for Crop Functional Genomics and Molecular Breeding, China Agricultural University, 2 West Yuanmingyuan Road, Beijing, 100193 P. R. China; 2 College of Agronomy/State Key Laboratory of Crop Biology, Shandong Agricultural University, Taian, 271018 P. R. China

**Keywords:** Maize, Grain water content, Grain dehydration rate, Physiological maturity, Mechanical harvesting

## Abstract

**Background:**

The low grain water content (GWC) at harvest is a prerequisite to mechanical harvesting in maize, or otherwise would cause massive broken kernels and increase drying costs. The GWC at harvest in turn depends on GWC at the physiological maturity (PM) stage and grain dehydration rate (GDR). Both GWC and GDR are very complex traits, governed by multiple quantitative trait loci (QTL) and easily influenced by environmental conditions. So far, a number of experiments have been conducted to reveal numbers of GWC and GDR QTL, however, very few QTL have been confirmed, and no QTL has been fine-mapped or even been cloned.

**Results:**

We demonstrated that GWCs after PM were positively correlated with GWC at PM, whereas negatively with GDRs after PM. With a recombinant inbred line (RIL) population, we identified totally 31 QTL related to GWC and 17 QTL related to GDR in three field trials. Seven GWC QTL were consistently detected in at least two of the three field trials, each of which could explain 6.92–24.78% of the total GWC variation. Similarly, one GDR QTL was consistently detected, accounting for 9.44–14.46% of the total GDR variation. Three major GWC QTL were found to overlap with three GDR QTL in bins 1.05/06, 2.06/07, and 3.05, respectively. One of the consistent GWC QTL, namely *qGwc1.1*, was fine-mapped from a 27.22 Mb to a 2.05 Mb region by using recombinant-derived progeny test. The *qGwc1.1* acted in a semi-dominant manner to reduce GWC by 1.49–3.31%.

**Conclusions:**

A number of consistent GWC and GDR QTL have been identified, and one of them, QTL-*qGwc1.1*, was successfully refined into a 2.05 Mb region. Hence, it is realistic to clone the genes underlying the GWC and GDR QTL and to make use of them in breeding of maize varieties with low GWC at harvest.

## Background

Maize is one of the most important crops in the world, serving as an essential source of feed, food, energy, and industrial raw materials. With the ever-increasing cost of human labor, mechanical harvesting of maize is the only choice in modern agriculture. Many developed countries, such as US and Germany, have fully achieved the mechanical harvesting in maize. However, the other countries, like China, have not yet achieved mechanical harvesting partly due to the shortage of suitable corn varieties [1]. To make mechanical harvesting feasible, maize varieties must have several excellent traits, such as disease resistance, lodging tolerance, and low grain water content (GWC) at harvest. The low GWC, for instance 15**–**25% at harvest, can protect the kernels against breakage during mechanical harvesting to benefit both grain yield and quality [1, 2]. However, this is not always the case. Taken China as an example, the GWC of maize hybrids ranges wildly from 25 to 40% at harvest, which severely limits the widespread application of mechanical harvesting [3]. Thus, reducing GWC at harvest has become a major goal for modern maize breeding in China.

Apart from broken kernels, high GWC causes many other problems. In warm and humid environments, for example, kernels with high GWC are more susceptible to ear sprouting, ear rot, and ear mold [4–7]. High GWC kernels can also delay the harvest and easily lead to ear dropping, plant lodging, and bird pecking [8, 9]. Even worse, high GWC kernels increase the costs associated with drying and storage [10, 11]. Farmers usually delay harvesting to reduce GWC. Nevertheless, the measure comes at the expense of delayed sowing time for the next season’s crops. Therefore, discovery of genetic factors controlling GWC could be the most cost-effective way for breeding maize cultivars with low GWC to reduce the kernel breakage and drying costs. The grain filling stage usually lasts for 45 days after pollination (DAP), and then the kernels enter a dehydration phase which usually takes another 15 days. The turning point between these two phases is called physiological maturity (PM), which is characterized by the disappearance of the milk line and formation of the black layer [12, 13]. PM is the critical stage to determine GWC at harvest. Water loss from kernels occurs in two phases. Before PM, the decrease in GWC is due to successive accumulation of dry matter via grain filling and the water loss rate is constant and highly dependent on genetic factors, which has been interpreted as a “developmental loss of water”. After PM, the accumulation of dry matter ceases, and the reduction in GWC is primarily due to water evaporation from kernels and thus can be greatly affected by environmental factors—the so-called “physical dehydration progress” [14, 15]. The low GWC at PM is indicative of the need to breed inbred lines with low GWC at harvest [16, 17]. On the other hand, the grain dehydration rate (GDR) before and after PM is also closely related to GWC at harvest [8, 18]. Maize varieties with a fast dry-down rate generally have low ear moisture at harvest [19, 20].

The GDR of maize is affected by many factors, such as variety, endosperm type, planting density, temperature, and humidity [21–23]. Compared with flint corns, dent corns have a higher GWC at PM, whereas tend to dry down faster after PM [24]. The fast grain filling rate but short filling duration is characteristic of fast GDR of maize variety [25, 26]. Moreover, the GDR will increase if a maize variety matures relatively early or if the growth period is relatively short [13, 19, 27–29]. The endosperm composition is critical to GDR, in that less hydrophilic compounds, such as sugars and water-soluble polysaccharides or more hydrophobic compounds promote GDR [30]. Low-oil hybrids have faster GDR after PM than high-oil hybrids [31]. Other agronomic traits also contribute to a relatively fast GDR; these include fewer kernel row number, a thin pericarp with high permeability [32–34], a relatively exposed ear-tip [35], lower water content in the husk and cob [36], a greater ear angle, and shorter cob length [37]. Premature death or senescence of corns or husks result in shorter, looser and thinner ears, which will promote GDR as well [28, 38, 39]. GDR correlates with air temperature when GWC is ≥30%; whereas GDR is associated with relative humidity when GWC is ≤30% [40].

Many studies have focused on understanding the genetic basis of GWC and GDR, both of which are complex quantitative traits. GDR was reported to exhibit a broad-sense heritability as high as 76.93% [41]. With the 181 F_2:3_ single-cross families, 10 GWC QTL and 8 GDR QTL were identified, totally accounting for 54.8–65.2% and 35.7–45.2% of the phenotypic variation, respectively [42]. By using 258 recombination inbred lines (RILs), as many as 40 GWC QTL and 35 GDR QTL were detected at four filling stages and a QTL on bins 5.03/04 could be considered as full-stage QTL [43]. With 232 RILs, 9 GDR QTL were detected after PM, each accounting for 5.77**–**13.63% of the total GDR variation, in which two QTL, namely *qKdr-2-1* and *qKdr-6-1*, were repeatedly detected in two environments [44]. In a similar work with 280 RILs, 14 GDR QTL were identified after PM, and each could explain 5.05**–**16.28% of the total GDR variation. Two of them, *qKdr-2-1* and *qKdr-3-6*, were consistently detected across both locations [45]. With 330 F_2:3_ families, 10 GWC at 45 DAP QTL, 10 GWC at harvest QTL and 10 AUDDC (area under the dry down curve) QTL were detected. Four of them, namely *q45dGM1–1*, *qHTGM2–2*, *qAUDDC2–1* and *qAUDDC10–1*, were stable across environments and could explain more than 10% of phenotypic variance [46]. In a genome-wide association study, 13 chromosomal segments significantly associated with GDR were identified, of which seven were located within the previously mapped GDR QTL [47]. By using 309 inbred maize lines, seven significant SNPs were repeatedly identified in four environments and six candidate genes were identified [48]. With Meta-analysis, eight meta-QTL were found to be associated with GWC at harvest [49]. Although many QTL have been identified, no QTL has been fine-mapped or even been cloned. Yet, the y allele that controls the white endosperm phenotype may have a pleiotropic effect on GWC at harvest [50].

In this study, we performed QTL analysis for GWC and GDR, and then carried out fine-mapping of a major QTL related to GWC that was consistently detected in three field trials. The results will enable cloning of the relevant gene within the QTL and its subsequent introduction via marker-assisted selection to reduce GWC and improve GDR in maize.

## Results

### Phenotypic analysis

**Fig. 1:**
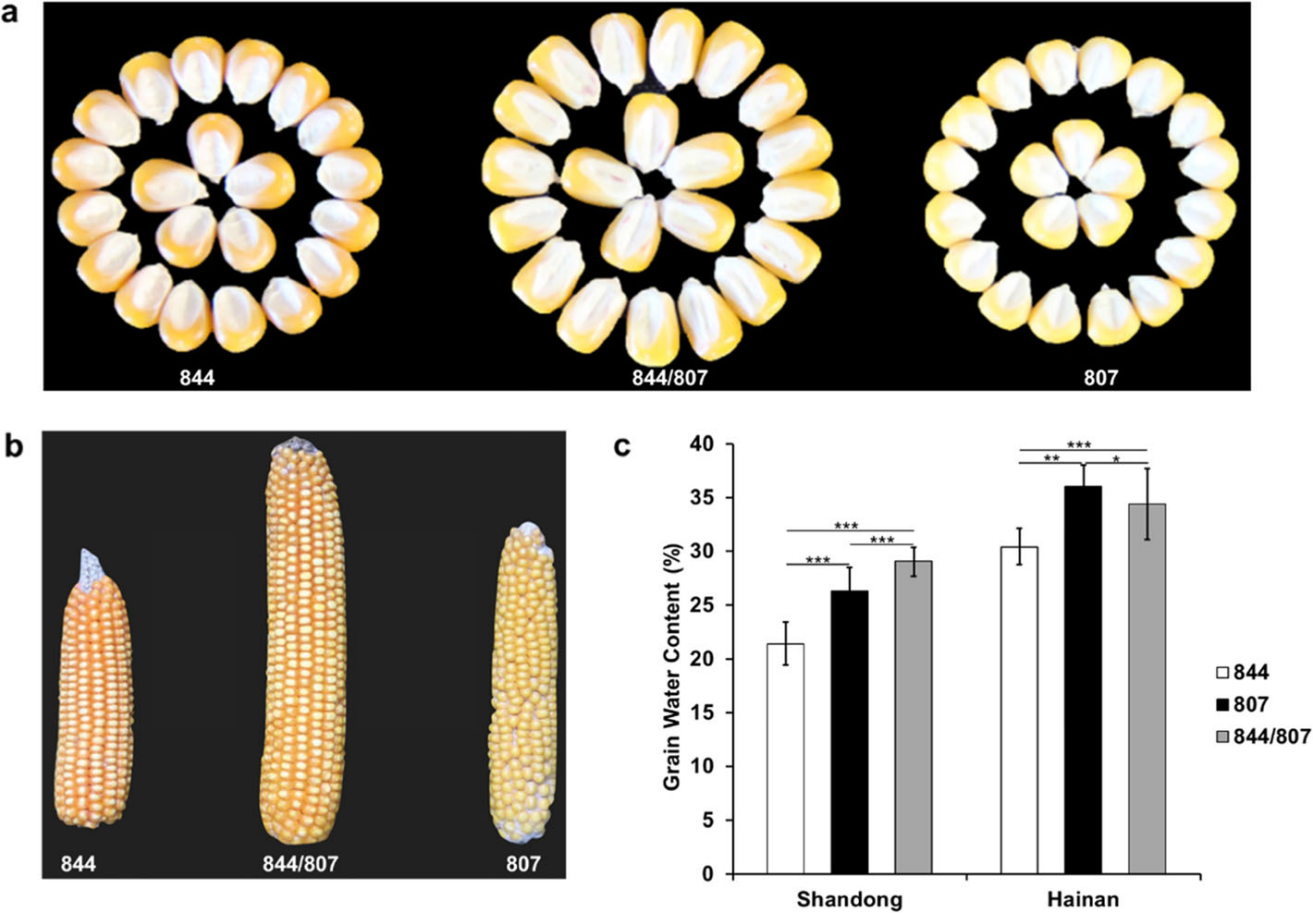
Comparison of morphological and GWC traits among parents “844”, “807” and their F1 hybrid “844/807”. **a** The comparison of kernel-size among “844”, “807” and “844/807”. **b** The comparison of ear-shape among “844”, “807” and “844/807”. **c** The GWC values of “844”, “807” and “844/807”. Values are GWC means ± SD. Statistical significance was determined using Student’s *t*-test: *P < 0.05, **P < 0.01, ***P < 0.001

**Fig. 2:**
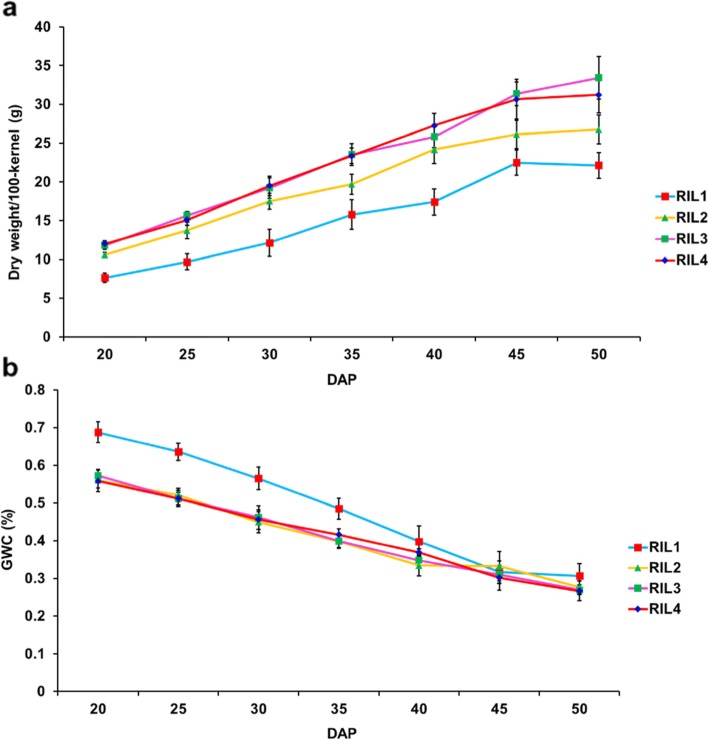
Grain dry weight/100-kernel and GWC values of the four RILs. Kernel samples were taken every 5 days, starting from 20 to 50 days after pollination (DAP). **a** The changes in grain dry weight/100-kernel during the grain-filling course. **b** The changes in GWC values during the grain-filling course. Values are dry weight/100-kernel ± SD (**a**) and GWC means ± SD (**b**)

Before QTL mapping, we compared the kernel-size and ear-shape of parental lines “844”, “807”, and their F_1_ hybrid “844/807”. These morphological traits are “844/807” > “844” > “807” in descending order (Fig. 1a, b). At the PM stage, the mean GWC values for “844”, “807” and “844/807” in Shandong were 21.44, 26.35 and 29.04%, and in Hainan the values were 30.42, 36.03 and 34.39%, respectively (Fig. 1c). We then selected four RILs to measure the dry weight/100-kernel and GWC every 5 days (seven times in total) starting from 20 DAP, which allowed us to monitor dynamic changes in grain filling and dehydration rate to determine the suitable sampling times. During the period of 20 to 45 DAP, the dry matter of each RIL accumulated linearly, while the GWC decreased synchronously (Fig. 2). Thereafter, four RILs differed with respect to the changing curves of dry weight/100-kernel and GWC. Of them, the RIL1 ceased an accumulation of the dry matter and slowed down the dehydration rate, while the other three RILs showed a continuous slow increase in the dry matter and a slow decrease in the GWC (Fig. 2).

During initial QTL mapping, kernels of each RIL were sampled twice (45 and 50 DAP, Shandong and Hainan in 2014) or four times (45, 50, 55, and 60 DAP, Shandong in 2015). Overall, the RIL population showed great variation in GWC, ranging from 10.93 to 59.50%, in the three field trials. The mean GWC values at different sampling times (i.e., DAP) were 39.37 and 31.62% in Shandong (summer, 2014), 27.61 and 18.87% in Hainan (winter, 2014), and 38.22, 32.78, 27.63 and 23.47% in Shandong (summer, 2015), respectively (Additional file 1: Table S1). The difference of GWC values from two successive samplings was used as the GDR, thus resulting in one GDR (45 to 50 DAP) in Shandong (2014) and Hainan (2014) and three GDRs in Shandong (2015): GDR1 (45 to 50 DAP), GDR2 (50 to 55 DAP), and GDR3 (55 to 60 DAP). The GWC and GDR distribution curves were characterized by typical quantitative traits (Additional file 2: Figure S1, Additional file 3: Figure S2). The analysis of variance (ANOVA) revealed that both GWC and GDR values differed significantly among RILs, environments, and interactions between RILs and environments at either 45 or 50 DAP. Broad-sense heritability (*h*^*2*^) was estimated as 74.80% for GWC and 38.36% for GDR (Additional file 4: Table S2, Additional file 5: Table S3).

We then conducted correlation analysis for different phenotypic data. The GWC values had the significant correlation coefficients (*r*) across the three field trials, ranging from 0.34 to 0.46; whereas the GDR had very low *r* values, ranging from 0.18 to 0.34 (Additional file 6: Table S4). The GWC at two sampling times showed significant *r* values in Shandong (2014) and Hainan (2014). Similarly, significant *r* values were found for GWC at four sampling times in Shandong (2015). However, the GDR values were not significantly correlated across the three periods after PM in Shandong (2015). Intriguingly, a significant negative correlation existed between GDR and GWC (later sampling) after PM in each of the three field trials (Table 1).

**Table 1:** Correlation analysis of GWC and grain dehydration rate (GDR) in three field trials

Locations	Traits	Shandong (2014)			Hainan (2014)			Shandong (2015)						
		GWC45	GWC50	GDR45-50	GWC45	GWC50	GDR45-50	GWC45	GWC50	GWC55	GWC60	GDR45-50	GDR50-55	GDR55-60
Shandong (2014)	GWC45^a^	1												
	GWC50^b^	0.58 **	1											
	GDR45-50	0.13	-0.74 ***	1										
Hainan (2014)	GWC45				1									
	GWC50				0.82 ***	1								
	GDR45-50				-0.04	-0.61 ***	1							
Shandong (2015)	GWC45							1						
	GWC50							0.74 ***	1					
	GWC55^c^							0.59 ***	0.73 ***	1				
	GWC60^d^							0.50 ***	0.71 ***	0.85 ***	1			
	GDR45-50^e^							0.21	-0.51 ***			1		
	GDR50-55^f^								-0.23*	-0.81 **		0.04	1	
	GDR55-60^g^									0.08	-0.44 ***	0.02	-0.12	1

^a^GWC45: sampled at 45 DAP

^b^GWC50: sampled at 50 DAP

^c^GWC55: sampled at 55 DAP

^d^GWC60: sampled at 60 DAP

^e^GDR45-50: the difference in GWC between 45 and 50 DAP

^f^GDR50-55: the difference in GWC between 50 and 55 DAP

^g^GDR55-60: the difference in GWC between 55 and 60 DAP

The values in the table indicate the correlation coefficient (*r*) and its significant difference: *P < 0.05, **P< 0.01, ***P < 0.001

### Initial QTL mapping of GWC and GDR

A genetic linkage map was constructed based on 129 RILs and 782 informative SNPs (Single Nucleotide Polymorphisms). The total genetic distance was 1522.48 cM with an average of 1.95 cM between adjacent markers (Additional file 7: Table S5, Additional file 8: Figure S3). For the purpose of QTL mapping, ears with low setting rates or no seeds were discarded. Ultimately, the phenotypic data selected from 84 (Shandong, 2014), 119 (Hainan, 2014), and 117 (Shandong, 2015) RILs were used for QTL mapping of GWC and GDR. For GWC, a total of 31 QTL were identified in the three field trials, including 11 in Shandong in 2014, 8 in Hainan in 2014, and 12 in Shandong in 2015. For GDR, 17 QTL were detected, including 5 in Shandong in 2014, 2 in Hainan in 2014, and 10 in Shandong in 2015. The GWC QTL were distributed in all maize chromosomes, and the GDR QTL were scattered on all maize chromosomes except chromosome 4. The phenotypic variation that can be explained by a single QTL ranged from 6.88 to 28.54%, with the LOD values ranging from 2.53 to 8.34. Of the 48 QTL, 27 trait-enhancing alleles (18 GWC and 9 GDR) were derived from the parent “844”, while other 21 trait-enhancing alleles (13 GWC and 8 GDR) were from the parent “807” (Additional file 9: Figure S4, Additional file 10: Figure S5, Additional file 11: Figure S6, Additional file 12: Table S6, Additional file 13: Table S7, Additional file 14: Table S8).

Two major QTL related to GWC, namely *qGwc1.1* and *qGwc3.2*, were consistently detected on bins 1.04/05 and 3.04/05 across three field trials, which could explain 10.32–24.47% and 6.92–15.74% of the total GWC variation, respectively. Other five QTL, namely *qGwc1.2*, *qGwc2.3, qGwc3.1*, *qGwc3.3* and *qGwc5.2*, were detected in any two of the three field trials. They were separately scattered on the five chromosomal bins 1.05/06, 2.06/07, 3.02/04, 3.05/06, and 5.05/06, accounting for 7.66–24.78%, 10.19–14.95%, 11.43–13.05%, 7.31–14.86%, and 8.33–13.97% of the total GWC variation, respectively. By contrast, only one major GDR QTL, *qGdr1.2* on bin 1.05/06, was consistently detected in Shandong in 2014 and 2015, and could explained 9.44–14.46% of the total GDR variation. Three GWC QTL, *qGwc1.2*, *qGwc2.3*, and *qGwc3.3*, were found to overlap with three GDR QTL, *qGdr1.2*, *qGdr2.3*, and *qGdr3.3*, respectively (Table 2). We are very interested in such QTL, like *qGwc1.2* and *qGdr1.2,* which exist in pairs and play synergic effects on reducing GWC and accelerating GDR to achieve low GWC at harvest. Apart from the QTL that were consistently detected as descried above, the other QTL could not be detected repeatedly and generally had a very small *R*^*2*^.

**Table 2:** The common QTL related to GWC and GDR in three field trials

QTL (bins)	Field trial	DAP	Rep	Flanking SNPs	Physical Location (Mb)	LOD	AE (%)	*R*^*2*^ (%)
*qGwc1.1* (1.04/1.05)	Shandong (2014)	50	R1	SYN3987-PZE-101101518	65.66–98.76	5.70	−2.68	16.23
			R2	SYN3987-PZE-101101518	65.66–98.76	2.78	−1.77	10.32
			AVE	SYN3987-PZE-101101518	65.66–98.76	6.53	−2.65	24.47
	Hainan (2014)	50	R1	SYN3987-PZE-101109414	65.66–117.16	5.68	−2.32	17.55
	Shandong (2015)	50	R1	SYN3987-PZA00944.1	65.66–89.00	3.93	−1.19	12.81
*qGwc1.2* (1.05/1.06)	Hainan (2014)	45	R1	PZA00944.1-PZE-101146598	89.00–189.77	8.34	−2.32	23.74
			R2	PZA00944.1-PZE-101146598	89.00–189.77	3.07	−1.26	7.66
			AVE	PZA00944.1-PZE-101146598	89.00–189.77	6.65	−1.74	17.50
		50	R2	PZA00944.1-PZE-101146598	89.00–189.77	7.81	−2.51	22.21
			AVE	PZA00944.1-PZE-101146598	89.00–189.77	6.98	−2.34	19.57
	Shandong (2015)	55	R1	PZA00944.1-PZE-101146598	89.00–189.77	3.72	−1.59	11.97
			AVE	PZA00944.1-PZE-101146598	89.00–189.77	2.68	−1.3	7.99
		60	R1	PZA00944.1-PZE-101146598	89.00–189.77	6.81	−2.63	24.78
			R2	PZA00944.1-PZE-101146598	89.00–189.77	3.03	−1.64	10.86
			AVE	PZA00944.1-PZE-101146598	89.00–189.77	4.75	−1.81	13.99
*qGwc2.3* (2.06/2.07)	Shandong (2014)	45	R2	PZE-102131962-SYN5428	182.34–190.16	2.85	1.33	10.19
	Shandong (2015)	50	R2	PZE-102131962-PZE-102145606	182.34–192.58	4.40	2.21	14.95
			AVE	SYN34721-PZE-102137972	177.75–186.79	4.45	1.50	14.02
*qGwc3.1* (3.02/3.04)	Shandong (2014)	45	AVE	PZE-103015388-PZE-103026528	8.27–19.65	2.67	1.34	11.43
	Shandong (2015)	45	R1	PZE-103014908-PZE-103022844	8.04–15.06	3.00	1.07	13.05
*qGwc3.2* (3.04/3.05)	Shandong (2014)	45	R1	PZE-103026528-PZE-103061612	19.65–106.24	4.37	1.57	15.74
			R2	PZE-103036305-PZE-103084178	29.80–139.51	2.53	1.34	8.54
		50	R1	PZE-103036305-PZE-103054563	29.80–63.69	3.13	3.90	15.55
	Hainan (2014)	45	R1	PZE-103033919-PZE-103082105	26.45–136.08	3.81	1.45	9.77
			R2	PZE-103033919-PZE-103082105	26.45–136.08	2.74	1.28	7.30
			AVE	PZE-103033919-PZE-103084178	26.45–139.51	5.30	1.60	13.39
		50	R1	PZE-103061612-PZE-103084178	106.24–139.51	3.69	1.90	10.31
	Shandong (2015)	45	AVE	PZE-103036305-PZE-103082105	29.80–136.08	2.97	0.87	9.33
		50	R1	PZE-103026528-PZE-103061612	19.65–106.24	2.88	1.04	9.10
		55	R1	PZE-103033919-PZE-103082105	26.45–136.08	2.67	1.37	8.54
		60	AVE	PZE-103036305-PZE-103084178	29.80–139.51	2.68	1.42	6.92
*qGwc3.3* (3.05/3.06)	Shandong (2014)	50	R1	PZE-103094339-PZE-103110355	155.32–170.68	2.93	2.98	11.29
			R2	PZE-103094339-PZE-103110355	155.32–170.68	3.01	1.91	11.63
			AVE	PZE-103087199-PZE-103110355	144.69–170.68	4.42	2.18	14.86
	Hainan (2014)	50	AVE	PZE-103094339-SYN31220	155.32–180.98	2.76	1.49	7.31
*qGwc5.2* (5.05/5.06)	Hainan (2014)	45	R1	SYN20663-SYN14995	181.89–203.82	4.90	−2.06	13.97
			AVE	SYN20663-SYN14995	181.89–203.82	2.92	−1.22	8.33
	Shandong (2015)	60	AVE	SYN7361-PZE-105137926	176.12–192.86	3.48	−2.00	10.23
*qGdr1.2* (1.05/06)	Shandong (2014)	45–50	R1	PZE-101093040-PZE-101135767	85.72–175.64	3.47	1.64	14.46
	Shandong (2015)	50–55	R1	PZE-101093040-PZE-101135767	85.72–175.64	2.94	1.09	11.38
			R2	PZA00944.1-PZE-101146598	89.00–189.77	2.95	1.16	9.44

A total of eight common QTL were detected, including seven GWC and one GDR QTL

Rep: indicates replications in each field trial, where “R1” and “R2” represent the first and second replication and “AVE” is the average value of R1 and R2. AE: the additive effect. *R*^*2*^: explained phenotypic variation

The best linear unbiased prediction (BLUP) model was used to predict GWC at 45 and 50 DAP across the three field trials. The predicted GWC and GDR were used as phenotypes to perform QTL mapping, and this identified four GWC and three GDR QTL. All seven QTL were found to overlap with those detected in the separate QTL analysis except for *qGdr8.1* on bin8.00/01 (Additional file 15: Table S9).

### Sequential fine-mapping of the major QTL-*qGwc1.1*

Based on the initial QTL mapping, we conducted high-resolution mapping of *qGwc1.1*, a consistent QTL responsible for reducing GWC. To develop the high-density markers within the 27.22 Mb interval of *qGwc1.1*, we re-sequenced both parents to search for sequence variations within *qGwc1.1*. In total, 17 SSR and 9 STS markers were developed to saturate the *qGwc1.1* region (Additional file 16 Table S10).

**Fig. 3:**
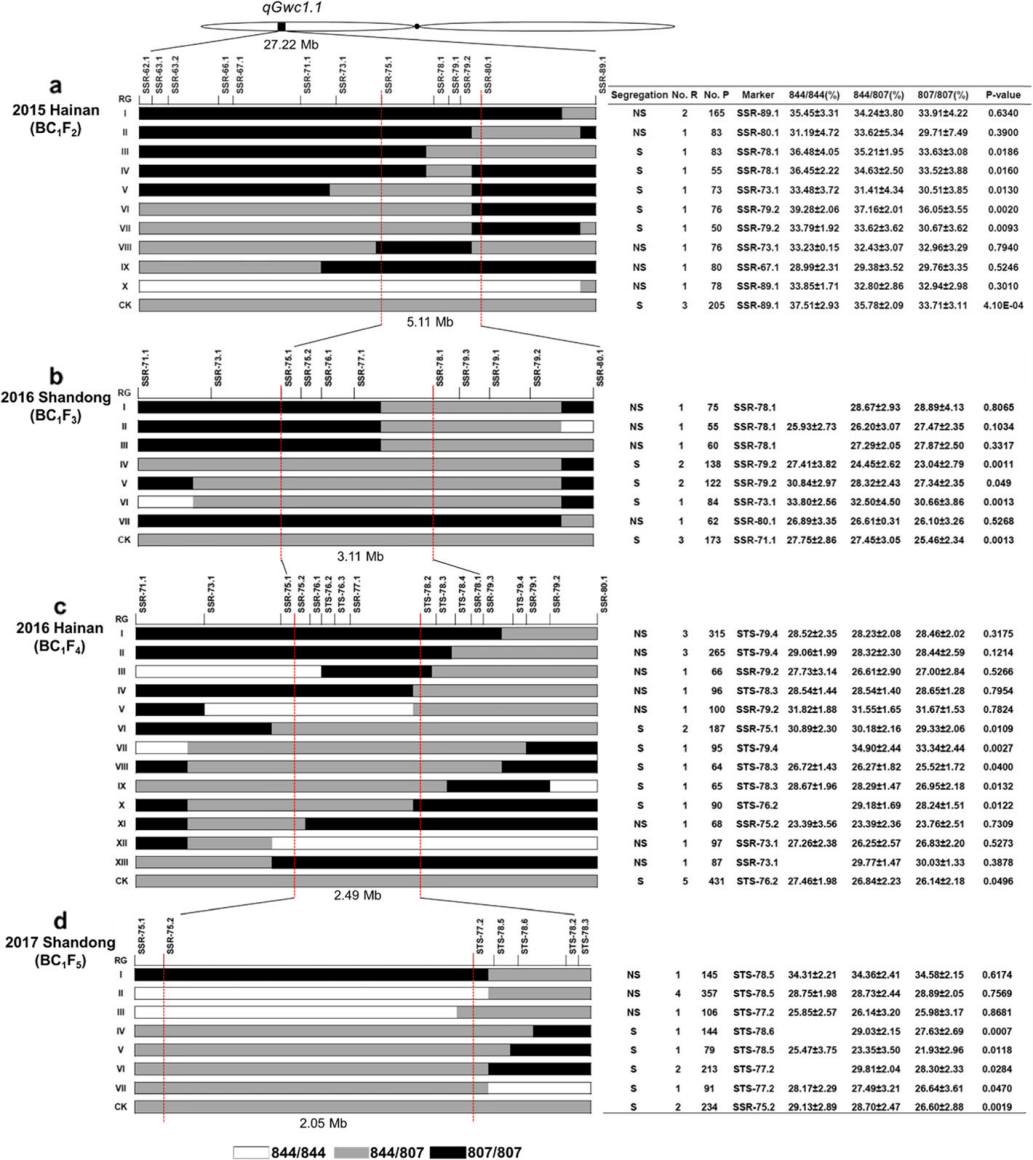
Sequential fine-mapping of the major QTL*-qGwc1.1*. **a** Fine-mapping was conducted in the winter of 2015 in Hainan. **b** Fine-mapping was conducted in the summer of 2016 in Shandong. **c** Fine-mapping was conducted in the winter of 2016 in Hainan. **d** Fine-mapping was conducted in the summer of 2017 in Shandong. For each recombinant genotype (RG), the chromosomal region at QTL*-qGwc1.1* is depicted as white (“844/844”), grey (“844/807”), and black (“807/807”) rectangles. Molecular markers in the heterozygous region were used to determine the genotypes of all progeny from each parental recombinant plant. The mean GWC values were calculated for various genotypes in the progeny. The *t*-test was used to determine the statistical significance of differences between GWC values among genotypes. In the right-side table, the heading “*P*-value*”* represents the difference between “844/844” and “807/807” in the self-cross populations and between “844/807” and “807/807” in the backcross populations. A *P*-value > 0.05 indicates that the GWC was not significantly different among genotypes and that the QTL-*qGwc1.1* did not exist within the heterozygous region. A *P*-value < 0.05 indicates that the GWC were significantly different among genotypes and that the QTL*-qGwc1.1* existed within the heterozygous region. The heading “Segregation” indicates whether the GWC significantly segregated or not among various genotypes within the progeny, where “S” denotes segregation (*P*-value < 0.05) and “NS” means non-segregation (*P*-value > 0.05). The heading “No. R” denotes the number of recombinants with the same genotype. The heading “No. P” denotes the number of plants. The heading “Marker” denotes the marker in the heterozygous region used for genotyping. The headings “844/844”, “807/807”, and “844/807” represent three different genotypes in the progeny. Four successive fine-mapping analyses narrowed the QTL interval from 22.72 Mb to 2.05 Mb

In the summer of 2015 in Beijing, we screened BC_1_F_2_ populations with markers SYN3987 and PZA00944.1, which flanked the mapped *qGwc1.1* region. The resultant 11 BC_1_F_2_ recombinants were then genotyped at 13 newly-developed SSR markers (SSR-62.1 to SSR-89.1), resulting in totally 10 distinct recombination types (Fig. 3a). In the winter of 2015 in Hainan, the recombinant-derived BC_1_F_3_ families, totaled 819 plants, were grown in the field. In order to obtain an unbiased estimate of genetic effect of *qGwc1.1*, the other BC_1_F_3_ family (205 plants), derived from three BC_1_F_2_ plants heterozygous at *qGwc1.1*, were also grown in the field as control. Each plant in the BC_1_F_3_ family was investigated for its genotype at *qGwc1.1* and GWC at PM. For a single BC_1_F_3_ family, the mean GWC values were calculated for three genotypes, i.e., the two homozygotes “844/844” and “807/807”, and one heterozygote “844/807”. A significant difference in average GWC between the two homozygotes would indicate the existence of *qGwc1.1*, while non-significant difference would imply the absence of *qGwc1.1* within the parental heterozygous region. This progeny test revealed significant differences in GWC for the types III–VII, but not I, II, and VIII–X. Upon comparison of all 10 recombination types to GWC performance in their progeny, *qGwc1.1* was delimited to an interval of markers SSR-75.1 and SSR-80.1, with the physical distance of 5.11 Mb as reference to the AGPv4 B73 genome sequence [51] (Fig. 3a).

In the newly mapped 5.11 Mb region, nine BC_1_F_3_ recombinants were identified, which were further self-pollinated or backcrossed to produce the next progeny. Accordingly, we designed four new SSR markers (SSR-75.2, SSR-76.1, SSR-77.1, and SSR-79.3) in the SSR-75.1/SSR-80.1 interval, which allowed us to categorize the nine BC_1_F_3_ recombinants into seven distinct types. Similarly, each recombinant-derived progeny, together with the heterozygote-derived control, was grown in the summer of 2016 in Shandong. A significant difference in GWC at PM between two homozygotes “844/844” and “807/807” was apparent for recombination types IV–VI, indicating the existence of *qGwc1.1* in their parental heterozygous region. However, this was not the case for the types II and VII in GWC between “844/844” and “807/807” in the self-pollinated progeny or the types I and III between “844/807” and “807/807” in the backcross progeny, suggesting the absence of *qGwc1.1* in their parental heterozygous region. Taken together, we further narrowed *qGwc1.1* to an interval of ~ 3.11 Mb (AGPv4), flanked by markers SSR-75.1 and SSR-78.1 (Fig. 3b).

In the winter of 2016 in Hainan, we designed five more STS markers (STS-76.1, STS-76.2, STS-78.2, STS-78.3 and STS-78.4) between SSR-75.1 and SSR-78.1 as well as an additional STS marker STS-79.4 downstream of SSR-78.1. These markers were used to genotype 18 new BC_1_F_4_ recombinants, resulting in 13 distinct recombination types. Significant differences in GWC in the progeny were observed for types VI–X, but not I–V and XI-XIII, suggesting the presence of *qGwc1.1* in the SSR-75.2/STS-78.2 interval with a physical distance of 2.49 Mb (AGPv4) (Fig. 3c).

We conducted the fourth fine-mapping in the summer of 2017 in Shandong. We developed three more STS markers (STS-77.2, STS-78.5 and STS-78.6) in the newly mapped 2.49 Mb region. With the newly-developed markers, the 11 recombinants obtained from BC_1_F_5_ progeny could be classified into seven types. Types I–III did not show significantly different GWC values among their progeny; whereas types IV–VII did show significant difference in GWC. These data further allowed us to narrow *qGwc1.1* to a 2.05 Mb interval flanked by markers SSR-75.2 and STS-77.2 (Fig. 3d).

### Genetic effect of *qGwc1.1* on GWC

**Fig. 4:**
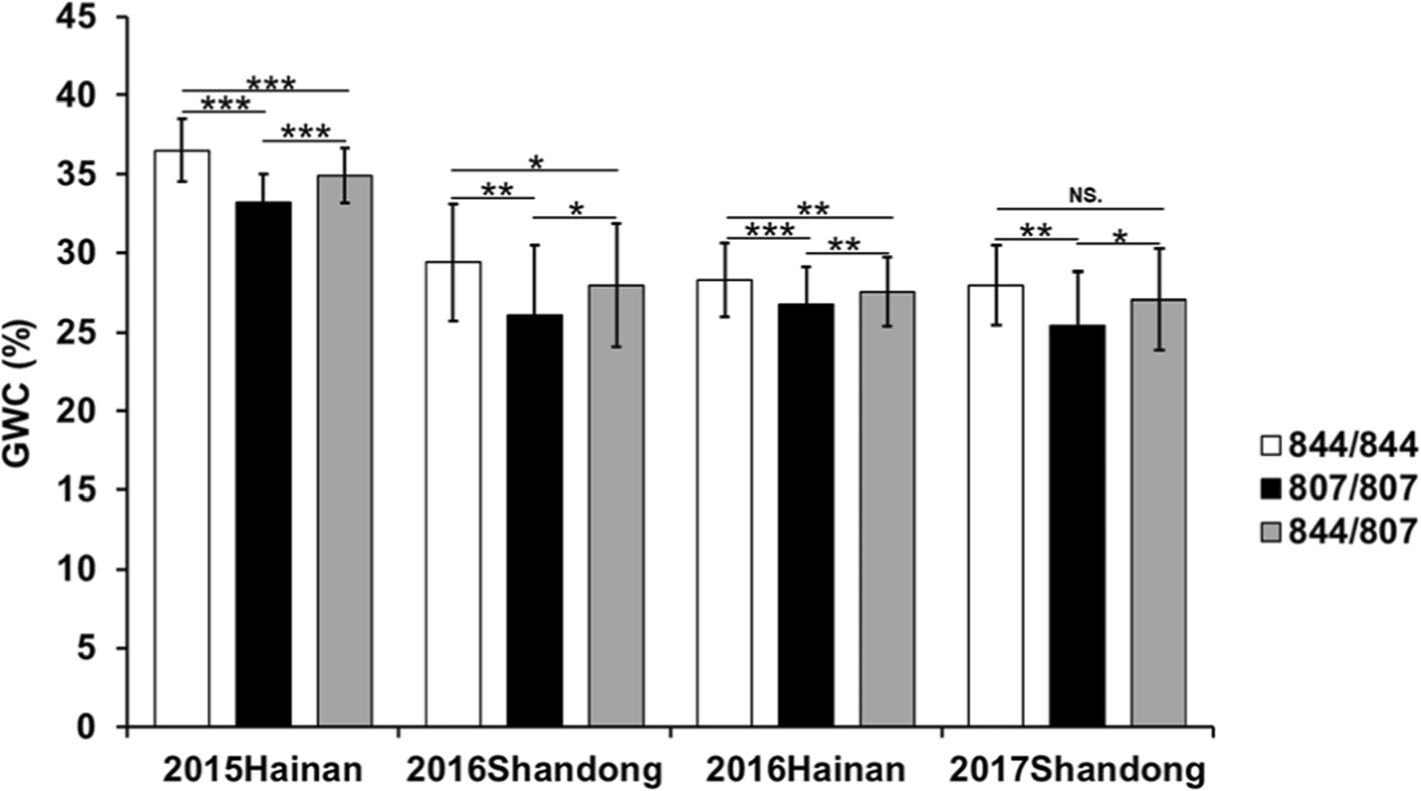
Evaluating the genetic effect of QTL*-qGwc1.1*. The mean GWC value was calculated for three genotypes (“844/844”, “807/807”, and “844/807”) in four rounds of fine-mapping efforts. Values are GWC means ± SD. Statistical significance was determined using Student’s *t*-test: *P < 0.05. *P < 0.05, **P < 0.01, ***P < 0.001

To evaluate the genetic effect of *qGwc1.1* on GWC, we investigated multiple self-pollinated progeny segregating at the *qGwc1.1* locus, including 542 BC_1_F_3_, 517 BC_1_F_4_, 932 BC_1_F_5_, and 823 BC_1_F_6_ plants. For the three genotypes “844/844”, “807/807”, and “844/807” in the segregating progeny, the respective mean GWC values were 36.5, 33.19, and 34.92% in the BC_1_F_3_ progeny, 29.39, 26.08, and 27.97% in the BC_1_F_4_ progeny, 28.30, 26.81, and 27.53% in the BC_1_F_5_ progeny, and 27.97, 25.44, and 27.06% in the BC_1_F_6_ progeny. The difference in GWC values was significant between the two homozygotes in each segregating progeny. The existence of *qGwc1.1* significantly reduced the GWC values by 3.31, 3.31, 1.49, and 2.53% in the BC_1_F_3_, BC_1_F_4_, BC_1_F_5_, and BC_1_F_6_ progeny, respectively (Fig. 4). The heterozygote “844/807” was found to have the median GWC value of the two homozygotes, indicating that *qGwc1.1* acted in a semi-dominant manner to reduce GWC in maize.

## Discussion

Development and deployment of maize varieties with low GWC at harvest is the premise of ensuring that mechanical harvesting can be applied. Discovery of genes related to GWC is crucial for understanding molecular mechanism that controls GWC and for breeding the low GWC varieties. For QTL mapping and cloning of GWC-related genes, the greatest challenge is how to obtain accurate GWC values since the GWC trait is controlled by multiple small-effect QTL and is easily influenced by environmental conditions. Many methods have been developed to measure GWC. For instance, the moisture determination metric via detection of variations in electric capacitance [52], the SK-300 moisture-determination meter [53], the digital timber-moisture meter (model BLD5601; General Electric Company, Lewinston, PA) [54], the wooden moisture meter Voltcraft FM-200 Humidity [55], the grain moisture meter with microwave attenuation at 10.5 GHz [56], and the hand-held moisture meter [17]. All these methods are effective, but require precise calibration. The classical oven-drying method, although laborious and time-consuming, is the most reliable way to obtain accurate GWC value [8, 26, 42, 45, 57, 58]. Considering that the mapping of a small-effect QTL requires extremely accurate GWC values, we finally chose an improved oven-drying method.

Considering that environmental factors can greatly affect GWC, we selected those RILs with similar flowering times to carry out pollination within a single day, and this guarantees that all plants were in sync with the grain filling and dehydration processes to reduce environmental errors. When sampling, we discarded those weakly-growing plants and low-setting ears to ensure that our data reflected samples of normal plants at the same developmental stage. Moreover, we sampled kernels in the middle part of ears, inactivated the enzymes, and then dried the kernels to a constant weight to ensure the complete loss of water. All these measures taken allowed us to obtain accurate GWC values for QTL mapping and fine-mapping.

The initial RIL population is consisting of 362 RILs. Due to environmental influences and individual differences, more than half of the RILs had either aging or no filaments when pollination. Consequently, only 84, 119 and 117 RILs were available in the three field trials, respectively, for an initial QTL mapping. The limited population size may result in larger confidence intervals, underestimation of QTL numbers, overestimation of QTL effects, and inability to quantify QTL interactions [59, 60], which makes it difficult to fine-map a QTL and estimate its genetic effect. In order to solve these problems, we performed three field trials with two replicates for each trial during QTL initial mapping, followed by fine-mapping of a consistent major QTL-*qGwc1.1*. After taking these measures, we did detect lots of consistent QTL, and narrowed down *qGwc1.1* from 27.22 Mb to 2.05 Mb. This indicates that the relatively small mapping population could be compensated for by multiple field trials with replicates in an aim to discover authentic QTLs.

It was reported that GWC at PM depends mainly on genetic factors and that GDR after PM is more likely to be influenced by environmental conditions [14, 15]. Our current results revealed significant correlations of GWC sampled at different time points, and the major GWC QTL at different sampling times overlapped with one another (Tables 1 and 2). At the PM stage, it has been postulated that grain filling ceases and that kernels enter a physical dehydration process [14, 15]. We therefore investigated the dynamic changes of GDR in the summer of 2015 and found that GDR values were negatively correlated with GWC values (later sampling) during three successive dehydration periods, namely GDR1 (45 to 50 DAP), GDR2 (50 to 55 DAP), and GDR3 (55 to 60 DAP), implicating that high GWC tends to slow down dehydration rate and *visa versa*. This was also observed when four RILs were used to investigate grain-filling rate and GWC over the entire course of grain development (Table 1, Fig. 2). The similar phenomenon was also observed by Li’s research [43]. All the findings demonstrated that it may be realistic to simultaneously improve both GWC and GDR.

The final GWC at harvest is related to both GWC at PM and GDR after PM. In the current study, we conducted a comprehensive QTL analysis of GWC and GDR at different filling stages, attempting to understand the genetic control of GWC during grain filling period. Of the GWC QTL detected, multiple QTL at different sampling times or from three field trials were overlapped. Similar result was observed for a single GDR QTL in the summer of 2014 and 2015. These findings suggest that the maize genome does have a set of genes responsible for GWC or GDR. We also detected certain consistent QTL for both GWC and GDR, suggesting that the same loci may underlie both GWC and GDR. For instance, *qGwc1.2* and *qGdr1.2* are localized in the same region, where *qGwc1.2* negatively affects GWC and *qGdr1.2* positively affects GDR. To our surprise, this locus has already been reported to control both GWC and GDR [43]. These findings suggest the existence of a potential allele at *qGwc1.2* (or *qGdr1.2*) that can reduce GWC at PM and meanwhile accelerate dehydration rate after PM, finally leading to low GWC at harvest. Besides, the finding that GWC is correlated negatively with GDR also supports the potential presence of such genes in the maize genome, which is similar with Qian’s research that the fast kernel dehydration lines have a lower moisture content at PM [53]. Surely, more researches are needed to reveal relevant genes responsible for either GWC or GDR or both from PM to harvest. In practice, genes that act simultaneously to reduce GWC and promote GDR are the most valuable for breeding of maize varieties with low GWC at harvest.

Comparing the QTL detected in the current study with those of other researchers, we found quite a number consistent QTL. For example, *qGwc1.2* is co-localized with the QTL found in two previous studies [4, 43]; *qGwc2.3* was detected by the other three groups [4, 61, 62]; *qGwc3.2* was also detected by another three groups [48, 63, 64], and the same situation is also observed for another 12 consistent QTL [65, 66] (Additional file 17: Table S11). All consistent QTL detected by multiple researches indicated that the existence of differential alleles responsible for GWC or GDR, which makes it possible to clone relevant genes in the future.

A consistent GWC QTL, *qGwc1.1*, was selected as the target QTL for fine-mapping. To reduce experimental error and elucidate the precise genetic effect of such a small-effect QTL, several measures have been taken. In four rounds of fine-mapping efforts, all progeny derived from a single recombinant were planted in a single experimental plot, and all individuals were pollinated at the same time. At the PM stage, all ears were sampled on a single day. To assess the genetic effect of *qGwc1.1*, we calculated an average GWC for each of the three genotypes in a given recombinant-derived progeny and evaluated the significant difference in GWC between two homozygotes. These data were then used to deduce whether the parental recombinant had *qGwc1.1* in its heterozygous region or not. With the availability of the recombination types and GWC values for all recombinants, we can narrow down the genomic region containing *qGwc1.1*. In the early generations, owing to genetic background noises, it was often difficult to obtain accurate GWC values. In advanced generations, however, plants in the same progeny had very similar genetic backgrounds and performed consistently in agronomic traits [67], and this enables us to unambiguously evaluate the genetic effect of *qGwc1.1*. Consequently, the *qGwc1.1* has been successfully narrowed from 27.22 Mb to 2.05 Mb, which could reduce GWC by 1.49–3.31% at PM. Future experimentations would focus on cloning the *qGwc1.1* gene and fine-mapping other seven consistent QTL to identify the underlying genes.

We found that two loci on chromosome 1, namely *qGwc1.1* and *qGwc1.2* (*qGdr1.2*), hold promise for reducing GWC via marker-assisted selection. They are adjacent QTL on bin1.04/06, *qGwc1.1* solely control GWC, whereas *qGdr1.2* affects both GWC and GDR, which implies the existence of a potential gene that could both reduce GWC and accelerate GDR after PM in *qGdr1.2*. The parent “807” has two elite alleles at both QTL that could be simultaneously introduced via marker-assisted selection. We have begun to introduce both alleles from the donor to elite maize varieties with high GWC values to breed maize varieties with low GWC. Considering that *qGwc1.1* acts in a semi-dominant manner, both parental lines with low GWC alleles would be the best choice for reducing GWC. This would require the use of different donors with a low GWC to improve both parental lines and thus avoid homozygous chromosomal region at the *qGwc1.1* in the F_1_ hybrid.

## Conclusions

Development and deployment of maize varieties with low GWC at harvest is one of the prerequisite conditions for mechanical harvesting. With the availability of a RIL population, we here detected a number of consistent GWC and GDR QTL. One of them, *qGwc1.1*, has been sequentially narrowed down into a 2.05 Mb interval by using recombinant-derived progeny. The *qGwc1.1* acted in a semi-dominant manner to reduce GWC by 1.49–3.31%. The current results make it possible to isolate the genes related to GWC and GDR and make use of them in development of maize varieties with low GWC at harvest for mechanical harvesting.

## Methods

### Plant materials

A maize RIL population from a cross between the inbred lines “844” and “807” was used in the current study. The line “844” was bred from a Reid heterotic group with low GWC, and “807” was from the P heterotic group with high GWC. The two parental inbred lines “844” and “807” as well as their RIL population were developed by Prof. Baoshen Liu of Shandong Agricultural University. The initial RIL population comprised 362 RILs. To ensure simultaneous pollination, we chose those RILs that had similar silking times in the field trails.

The field tests were repeated for three times. In the summer of 2014, we planted 94 RILs in the experimental station of Shandong Agricultural University (Taian, Shandong, E 117°06′, N 36°11′). In the winter of 2014, we planted 134 RILs (including the 94 RILs aforementioned) in the Hainan winter nursery (Jiusuo, Hainan, E108°54′, N 18°26′). In the summer of 2015, we planted the same 134 RILs in the experimental station of Shandong Agricultural University. To accelerate the fine-mapping process, we backcrossed each of the 134 RILs with the recurrent parent “807” in the winter of 2014. The resultant BC_1_F_1_ lines were self-crossed to produce the BC_1_F_2_ family in the spring of 2015 in the experimental station of China Agricultural University (Shangzhuang, Beijing). Those BC_1_F_2_ families contained the target QTL region were used for fine-mapping. Thereafter, recombinants screened from the newly-mapped QTL region were self-pollinated to produce progeny for further fine-mapping. From the winter of 2015 to the summer of 2017, we continuously performed the fine-mapping twice each year to narrow the target QTL by using BC_1_F_2_ to BC_1_F_5_.

### Field planting

For the initial QTL mapping, we implemented a completely randomized plot design with two replicates for each of three field trials. We planted 17 kernels in a 4.0 m row, and the distance between two adjacent plants was 0.25 m and the row spacing was 0.80 m. For the fine-mapping effort, each individual plot contained plants grown from seeds of a single recombinant-derived progeny.

### Uniform pollination

Parchment paper bags were used to prevent ears from pollination. Considering the pollen duration of RILs was different, RILs couldn’t be pollinated with the fresh pollens of themselves at the same day. In order to solve this problem, fresh ample pollens from the recurrent parent “807” were collected to simultaneously pollinate all RILs within a single day. Similarly, we conducted pollination for a given recombinant-derived progeny when 90% plants were silking, which ensured all ears within the same population were synchronized with respect to developmental stage to minimize any environmental influence on grain-filling and dehydration.

### Measurement of GWC

Four RILs were selected to elucidate the dynamic changes of GWC and GDR during the whole grain development. One hundred kernels were sampled every 5 days for a total of seven times, starting from 20 days after pollination (DAP). For each sample, grain fresh weight was immediately measured, followed by air-drying to constant weight (i.e., grain dry weight). GWC was calculated by the following formula:

GWC (%) = (grain fresh weight – grain dry weight)/grain fresh weight× 100%.

For QTL mapping, GWC was measured every 5 days for two or four times, starting from 45 DAP when kernels reached PM. We harvested three to four ears per RIL with intact husks and put them into an airtight plastic bag in the field. We then peeled off the husks in the lab, collected the kernels in the middle part of ears for about 100 kernels, and then mixed all the kernels collected for each particular RIL. The mixed kernels from a single RIL were immediately weighed to obtain the fresh weight. The kernels were placed in a craft paper bag and dried in a forced-hot-air dryer at 105 °C for 10 min to stop oxidative respiration via the inactivation of enzymes, followed by drying at 80 °C to a constant weight. The dried kernels were weighed to obtain grain dry weight. Those faint individuals and low-setting ears were discarded to ensure that our data reflected samples taken from normal plants at the same developmental stage. GWC was calculated by the same formula aforementioned and GDR was calculated by the following formula:

GDR (%) = GWC (early sampling) – GWC (later sampling).

For the fine-mapping efforts, however, we just measured GWC once at PM for a given recombinant-derived progeny.

### Analysis of phenotypic data

Statistical analyses were performed with the Microsoft Excel 2016 and R project [68]. We drew the line chart of dry weight/100-kernel and GWC of the four RILs, and the frequency distribution histogram and normal distribution curve for both GWC and GDR in three field trials. We performed ANOVA (part of the R project) to assess the effects of genotypes, environments, sampling times and the interactions between genotypes and locations on the phenotypic performance. The correlation coefficient (*r*) was calculated for GWC and GDR across three field trials. Moreover, a correlation analysis of GWC and GDR sampled at different time points was performed for each field trial.

We built an ANOVA model as follows: *Y*_*ijk*_ = *μ* + *G*_*i*_ + *E*_*j*_ + *S*_*k*_ + *G* × *E*_*ij*_ + *ε*_*ijk*_, in which *i* = 1, 2,.., *n*; *j* = 1, 2,.., *l*; *k* = 1, 2,.., *r*, here *n* is the number of RILs, *l* is the number of environments and *r* is the number of sampling times in the ANOVA of GWC whereas it is the number of replication in the ANOVA of GDR. Further *Y*_*ijk*_ is the observed value, *μ* is the population average value, *G*_*i*_ is the genetic effect of *i*th RIL, *E*_*j*_ is the environment effect of *j*th location, *S*_*k*_ is the sampling effect of *k*th sample, *G* × *E*_*ij*_ is the effect of *i*th gene and *j*th environment interaction, and *ε*_*ijk*_ is the random error. We also used the ANOVA result to calculate the broad-sense heritability (*h*^*2*^) using the formula $:{h}^2={\sigma}_G^2/\left({\sigma}_G^2+{\sigma}_{G\times E}^2/l+{\sigma}_e^2/ lr\right)$, where ${\sigma}_G^2$ is the genetic variance, ${\sigma}_{G\times E}^2$ is the variance of the interaction between genes and the environments, and ${\sigma}_e^2$ is the variance of the random error.

Student’s *t*-test was performed in QTL fine-mapping study to compare the difference in GWC between the “844/844” and “807/807” genotypes in self-pollinated populations or between the “844/807” and “807/807” genotypes in backcrossed populations.

### Construction of a linkage map and QTL detection

The RILs were genotyped with an Illumina GoldenGate 3KSNP chip. The raw data were filtered for the construction of genetic linkage map. SNPs that were polymorphic between two parental lines were chosen to calculate the missing rate and heterozygous rate. A SNP was deleted if the missing or heterozygous rate was more than 20%. We constructed the genetic linkage map using the maximum likelihood mapping method of the program JoinMap4.0 [69], followed by chi-square test to exclude those markers with segregation distortion. The Kosambi mapping method [70] was used to calculate the genetic distances between two adjacent SNPs. QTL mapping was performed with Windows QTL Cartographer2.5 [71] with the composite interval mapping method [72]. We set up the QTL detection parameters with the 1000-permutation test and with a logarithm of odds (LOD) score of > 2.5 to affirm a putative QTL. Any QTL with an explained phenotypic variation (*R*^*2*^) of > 10% was defined as a major QTL.

An initial QTL mapping was conducted for each of the three field trials, in which two replicates were analyzed separately or integrally to detect all potential QTL. Moreover, BLUP method was used to predict expectations of GWC and GDR for each RIL across three field trials to perform QTL mapping [73]. The GWC phenotypic data were analyzed using the statistical model:

*y*_*ijk*_ = *μ* + *g*_*i*_ + *l*_*j*_ + *r*_*k*_(*l*_*j*_) + (*g* × *l*)_*ij*_ + *ε*_*ijk*_, all of the factors are random effects, in which *y*_*ijk*_ is the observed value for the *i*th genotype in the *k*th replicate in *j*th environment, *μ* is the overall average value, *g*_*i*_ is the genetic effect of *i*th RIL, *l*_*j*_ is the environment effect of *j*th location, *r*_*k*_(*l*_*j*_) is the effect of *k*th replicate in the *j*th location, (*g* × *l*)_*ij*_ is the effect of *i*th genotype and *j*th environment interaction and *ε*_*ijk*_ is the residual. We calculated the excepted GWC of BLUP at 45 and 50 DAP. Then we calculated GDR of BLUP by the difference of the GWC of BLUP at 45 and 50 DAP.

### Development of polymorphic markers and genotyping

A set of molecular markers was developed based on sequence differences in the mapped regions, including simple sequence repeats (SSR) and sequence-tagged site (STS). To develop SSR markers, we downloaded the maize B73 reference sequence [51] and used the software SSRHunter1.3 to search for SSRs within the mapped regions. The primers flanking SSRs were designed with two tools, namely the primer3.0 [74] and the Primer Quest Tool [75]. To develop STS markers, two parental lines were subjected to whole genome-resequencing by NovoGene to search for insertions/deletions to design flanking primers within the mapped region. Then, we compared primer sequences to test their specificities for sites in the maize genome BLAST search [76]. The primer lengths range from 18 to 23 bp with an annealing temperature of 55°C or 60°C. Leaves at the eight-leaf stage were sampled, and then DNA was extracted using the SDS method [77]. PCR products were subjected to electrophoresis on an agarose or polyacrylamide gel to check for polymorphism. The DNA bands representing PCR products were labelled as follows: “1” if a band was the same as that of the parent “844”, “2” if the same as that of the parent “807”, or “3” if there were two bands—one from “844” and one from “807”. If the genotypes of two adjacent markers were different, then it could be considered a recombination breakpoint exist between the two markers.

### Sequential fine-mapping based on recombinant-derived progeny test

The RILs with recombination within the target QTL were backcrossed to two parents to produce progeny (BC_1_F_1_). Then the BC_1_F_1_ plants were self-pollinated to produce progeny (BC_1_F_2_) or backcrossed again to two parents to produce progeny (BC_2_F_1_). All progeny derived from a single parental recombinant RIL were grown in the same plot to investigate both genotypes and phenotypes (GWC at PM) for all plants. In the self-pollinated progeny, there were three genotypes: homozygote with the donor QTL region, heterozygote, and homozygote without the donor QTL region. In the backcross progeny, there are two genotypes: heterozygote and homozygote. The mean values of different genotypes in the same progeny were calculated and used to test statistical differences among various genotypes with a paired-sample *t*-test. Significant difference in GWC (*P* ≤ 0.05) between two homozygous genotypes or between homozygous and heterozygous genotypes indicates the presence of the QTL in the donor segment, or otherwise the absence of the QTL in the donor segment. Furthermore, the availability of genotypes of all progeny enables us to select new recombinants within the newly-mapped QTL region for the next round of fine-mapping process [78–80]. Additional file 18 shows the experimental flow chart.

## Supplementary information

**Additional file 1: Table S1.** Number of samples and average GWC in the three field trials. The number of samples related to the sampling time. The average GWC values for different sampling times in three field trials.

**Additional file 2: Figure S1.** Histogram of the frequency distribution and probability density curve of GWC values for the RILs in the three field trials. A-B, GWC values for the first and second samplings at 45 (A) and 50 DAP (B) in the summer of 2014 in Shandong. C-D, GWC values for the first and second samplings at 45 (C) and 50 (D) DAP in the winter of 2014 in Hainan. E-H, GWC values for the four samplings at 45 (E), 50 (F), 55 (G), and 60 (H) DAP in the summer of 2015 in Shandong. The GWC values on the *x* axis denote the boundary values for defining the GWC groups. The *y* axis on the left denotes the numbers of RILs.

**Additional file 3: Figure S2.** Histogram of the frequency distribution and probability density curve of GDR values for the RILs in the three field trials. A-B, GDR values at 45–50 DAP in 2014 in Shandong (A), 2014 in Hainan (B). C-E, GDR values in 2015 in Shandong at 45–50 DAP (C), 50–55 DAP (D), and 55–60 (E). The GDR values on the *x* axis denote the boundary values for defining the GDR groups. The *y* axis on the left denotes the numbers of RILs related to the GDR groups.

**Additional file 4: Table S2.** Analysis of variance of GWC in three field trials. Sources: variation sources. *df*: degrees of freedom. SS: sum of squares. MS: mean squares. EMS: estimated mean square. *P*-value: significant difference among sources. *P < 0.05, **P < 0.01, ***P < 0.001.

**Additional file 5: Table S3.** Analysis of variance of GDR in three field trials. Sources: variation sources. *df*: degrees of freedom. SS: sum of squares. MS: mean squares. EMS: estimated mean square. *P*-value: significant difference among sources. *P < 0.05, **P < 0.01, ***P < 0.001.

**Additional file 6: Table S4.** Correlation coefficient (*r*) for GWC and GDR between any two of three initial mapping trials. The correlation analysis was performed using the GWC at 45 and 50 DAP, as well as the GDR sampled at 45–50 DAP. GWC 45 DAP: GWC measured at 45 DAP. GWC 50 DAP: GWC measured at 50 DAP. GDR 45–50 DAP: GDR measured at 45–50 DAP. 14SD: The summer of 2014 in Shandong. 14HN: The winter of 2014 in Hainan. 15SD: The summer of 2015 in Shandong. The values in the table indicate the correlation coefficient (*r*) and its significant difference: *P < 0.05, **P < 0.01, ***P < 0.001. The correlation analysis was performed using the GWC at 45 and 50 DAP, as well as the GDR sampled at 45–50 DAP.

**Additional file 7: Table S5.** Marker and genetic distance information for the 10 maize linkage groups. Chr.: chromosome; No. of Markers: number of markers on each chromosome.

**Additional file 8: Figure S3.** Ten maize genetic linkage groups.

**Additional file 9: Figure S4.** Initial QTL mapping for GWC in three field trials. A, LOD profiles (upper) and additive genetic effects (lower) of ten maize chromosomes. B, QTL on chromosome 1. The legend with different lines and colors to the right indicates the sources of GWC. 1–6: GWC measured at 45 DAP with two replications (1–2) and their average value (3) and 50 DAP with two replications (4–5) and their average value (6) in Hainan in 2014. 7–12: GWC measured at 45 DAP with two replications (7–8) and their average value (9) and 50 DAP with two replications (10–11) and their average value (12) in Shandong in 2014. 13–24: GWC measured in Shandong in 2015, where 13–15 are two replications sampled at 45 DAP and their average value, 16–18 are two replications sampled at 50 DAP and their average value, 19–21 are two replications sampled at 55 DAP and their average value 22–24 are two replications sampled at 60 DAP and their average value. The *x* axes of both figures represent the genetic distance of different chromosomes. The *y* axis (upper) represents the LOD values for the QTL. The *y* axis (lower) represents the additive values for the QTL.

**Additional file 10: Figure S5.** Initial QTL mapping results for GDR in three field trials. A, LOD profiles (upper) and additive genetic effects (lower) of ten maize chromosomes. B, QTL on chromosome 1. The legend with different lines and colors to the right indicates the sources of GDR. 1–2: GDR of two replications measured in Hainan in 2014. 3–4: GDR of two replications measured in Shandong in 2014. 5–7: GDR measured at 45–50 DAP, 50–55 DAP and 55–60 DAP from the first replication in Shandong in 2015. 8–10: GDR measured at 45–50 DAP, 50–55 DAP and 55–60 DAP from the second replication in Shandong in 2015. The *x* axes of both figures represent the genetic distance of different chromosomes. The *y* axis (upper) represents the LOD values for the QTL. The *y* axis (lower) represents the additive values for the QTL.

**Additional file 11: Figure S6.** Initial QTL mapping results for GWC and GDR from BLUP analysis. LOD profiles (upper) and additive genetic effects (lower) of ten maize chromosomes. The legend with different colors to the right indicates GWC at 45 and 50 DAP as well as GDR calculated by BLUP. The *x* axes of both figures represent the genetic distance of different chromosomes. The *y* axis (upper) represents the LOD values for the QTL. The *y* axis (lower) represents the additive values for the QTL.

**Additional file 12: Table S6.** Initial QTL mapping of GWC and GDR in the summer of 2014 in Shandong. Traits: the phenotypes. QTL: the names of QTL which were detected in the initial QTL mapping. Sources: the sources of phenotypes, 14sd1–1 and 14sd1–2 represent the phenotypes sampled at 45 DAP of replicate 1 and replicate 2 in the summer of 2014 in Shandong, respectively; 14sd2–1 and 14sd2–2 represent the phenotypes sampled at 50 DAP of replicate 1 and replicate 2 in the summer of 2014 in Shandong, respectively; 14sd-ave1 and 14sd-ave2 represent the average phenotypes sampled at 45 and 50 DAP, respectively. 14sddr1 and 14sddr2 represent the GDR between 45 and 50 DAP of replicate 1 and replicate 2, respectively. Rep: the names of replications related to phenotypic data. “AVE”: represents the phenotypes from the average value of R1 and R2. DAP: Days after pollination. Bins: the location of the QTL in the chromosomes. Flanking SNPs: the SNPs at the both sides of QTL. Physical Location (Mb): the physical location of the QTL. CI (Mb): size of confident interval. AE: additive effect. *R*^*2*^: explained phenotypic variation.

**Additional file 13: Table S7.** Initial QTL mapping of GWC and GDR in the winter of 2014 in Hainan. Traits: the phenotypes. QTL: the names of QTL which were detected in the initial QTL mapping. Sources: the sources of phenotypes, 14hn1–1 and 14hn1–2 represent the phenotypes sampled at 45 DAP of replicate 1 and replicate 2 in the winter of 2014 in Hainan, respectively; 14hn2–1 and 14hn2–2 represent the phenotypes sampled at 50 DAP of replicate 1 and replicate 2 in the winter of 2014 in Hainan, respectively; 14hn-ave1 and 14hn-ave2 represent the average phenotypes sampled at 45 and 50 DAP, respectively. 14hndr1 and 14hndr2 represent the GDR between 45 and 50 DAP of replicate 1 and replicate 2, respectively. Rep: the names of replications related to phenotypic data. “AVE”: represents the phenotypes from the average value of R1 and R2. DAP: Days after pollination. Bins: the location of the QTL in the chromosomes. Flanking SNPs: the SNPs at the both sides of QTL. Physical Location (Mb): the physical location of the QTL. CI (Mb): size of confident interval. AE: additive effect. *R*^*2*^: explained phenotypic variation.

**Additional file 14: Table S8.** Initial QTL mapping of GWC and GDR in the summer of 2015 in Shandong. Traits: the phenotypes. QTL: the names of QTL which were detected in the initial QTL mapping. Sources: the sources of phenotypes, 15sd1–1 and 15sd1–2 represent the phenotypes sampled at 45 DAP of replicate 1 and replicate 2 in the summer of 2015 in Shandong, respectively; 15sd2–1 and 15sd2–2 represent the phenotypes sampled at 50 DAP of replicate 1 and replicate 2 in the summer of 2015 in Shandong, respectively; 15sd3–1 and 15sd3–2 represent the phenotypes sampled at 55 DAP of replicate 1 and replicate 2 in the summer of 2015 in Shandong, respectively; 15sd4–1 and 15sd4–2 represent the phenotypes sampled at 60 DAP of replicate 1 and replicate 2 in the summer of 2015 in Shandong, respectively; 15sd-ave1, 15sd-ave2, 15sd-ave3 and 15sd-ave4 represent the average phenotypes sampled at 45, 50, 55 and 60 DAP, respectively. 15sddr1–2-1, 15sddr1–2-2 represent the GDR between 45 and 50 DAP of replicate 1 and replicate 2, respectively. 15sddr2–3-1, 15sddr2–3-2 represent the GDR between 50 and 55 DAP of replicate 1 and replicate 2, respectively. 15sddr3–4-1, 15sddr3–4-2 represent the GDR between 55 and 60 DAP of replicate 1 and replicate 2, respectively. Rep: the names of replications related to phenotypic data. “AVE”: represents the phenotypes from the average value of R1 and R2. DAP: Days after pollination. Bins: the location of the QTL in the chromosomes. Flanking SNPs: the SNPs at the both sides of QTL. Physical Location (Mb): the physical location of the QTL. CI (Mb): size of confident interval. AE: additive effect. *R*^*2*^: explained phenotypic variation.

**Additional file 15: Table S9.** Initial QTL mappings of GWC and GDR from BLUP analysis in three field trials. DAP: Days after pollination. Bins: the location of the QTL in the chromosomes. Flanking SNPs: the SNPs at the both sides of QTL. Physical Location (Mb): the physical location of the QTL. CI (Mb): size of confident interval. AE: additive effect. *R*^*2*^: explained phenotypic variation.

**Additional file 16: Table S10.** Molecular markers developed in the QTL-*qGwc1.1* region on chromosome 1.

**Additional file 17: Table S11.** Comparison of the QTL detected in the current study with those revealed by other researchers.

**Additional file 18: Figure S7.** Experimental flow chart for identifying QTL and fine-mapping.

## Data Availability

Datasets used in the current study are available from the corresponding author on reasonable request.

## Abbreviations

ANOVA: Analysis of variance
AUDDC: Area under the dry down curve
BLUP: Best linear unbiased prediction
GDR: Grain dehydration rate
GWC: Grain water content
PM: Physiological maturity
QTL: Quantitative trait loci
SNP: Single nucleotide polymorphism
