# Supplementary material for: Genetic dissection of grain water content and dehydration rate related to mechanical harvest in maize
Source: BMC Plant Biol. 2020 Mar 17;20:118. doi: 10.1186/s12870-020-2302-0 (PMC7076969; doi:10.1186/s12870-020-2302-0)
Supplement: Supplementary file 1 — Additional file 1: Table S1. Number of samples and average GWC in the three field trials. The number of samples related to the sampling time. The average GWC values for different sampling times in three field trials. [file 12870_2020_2302_MOESM1_ESM.docx]

**Table S1** Number of samples and average GWC in the three field trials

| **Year** | **Location** | **Sampling Time (DAP)** | **No. of Samples** | **Average GWC (%)** |
| --- | --- | --- | --- | --- |
| 2014 | Shandong | 45 | 84 | 39.37 |
|  |  | 50 | 82 | 31.62 |
|  | Hainan | 45 | 118 | 27.61 |
|  |  | 50 | 119 | 18.87 |
| 2015 | Shandong | 45 | 109 | 38.22 |
|  |  | 50 | 110 | 32.78 |
|  |  | 55 | 109 | 27.63 |
|  |  | 60 | 106 | 23.47 |

**No. of Samples**: the number of available samples related to the sampling time.

**Average GWC**: the average GWC values for different sampling times in three field trials.
