# Supplementary material for: Genetic dissection of grain water content and dehydration rate related to mechanical harvest in maize
Source: BMC Plant Biol. 2020 Mar 17;20:118. doi: 10.1186/s12870-020-2302-0 (PMC7076969; doi:10.1186/s12870-020-2302-0)
Supplement: Supplementary file 2 — Additional file 2: Figure S1. Histogram of the frequency distribution and probability density curve of GWC values for the RILs in the three field trials. A-B, GWC values for the first and second samplings at 45 (A) and 50 DAP (B) in the summer of 2014 in Shandong. C-D, GWC values for the first and second samplings at 45 (C) and 50 (D) DAP in the winter of 2014 in Hainan. E-H, GWC values for the four samplings at 45 (E), 50 (F), 55 (G), and 60 (H) DAP in the summer of 2015 in Shandong. The GWC values on the x axis denote the boundary values for defining the GWC groups. The y axis on the left denotes the numbers of RILs. [file 12870_2020_2302_MOESM2_ESM.docx]

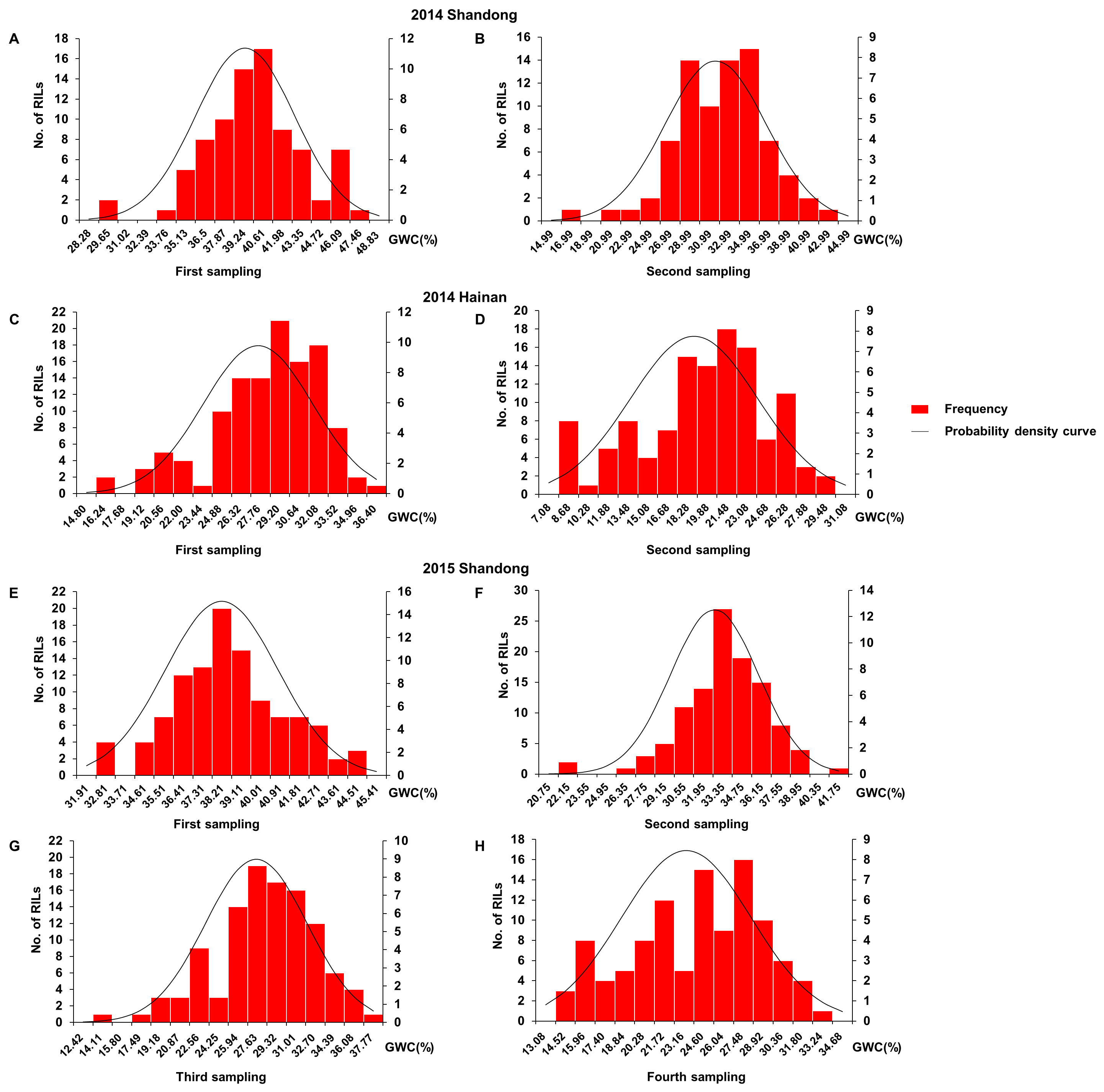


**Figure S1** Histogram of the frequency distribution and probability density curve of GWC values for the RILs in the three field trials.

A-B, GWC values for the first and second samplings at 45 (A) and 50 DAP (B) in the summer of 2014 in Shandong. C-D, GWC values for the first and second samplings at 45 (C) and 50 (D) DAP in the winter of 2014 in Hainan. E-H, GWC values for the four samplings at 45 (E), 50 (F), 55 (G), and 60 (H) DAP in the summer of 2015 in Shandong. The GWC values on the *x* axis denote the boundary values for defining the GWC groups. The *y* axis on the left denotes the numbers of RILs.
