# Supplementary material for: Genetic dissection of grain water content and dehydration rate related to mechanical harvest in maize
Source: BMC Plant Biol. 2020 Mar 17;20:118. doi: 10.1186/s12870-020-2302-0 (PMC7076969; doi:10.1186/s12870-020-2302-0)
Supplement: Supplementary file 3 — Additional file 3: Figure S2. Histogram of the frequency distribution and probability density curve of GDR values for the RILs in the three field trials. A-B, GDR values at 45–50 DAP in 2014 in Shandong (A), 2014 in Hainan (B). C-E, GDR values in 2015 in Shandong at 45–50 DAP (C), 50–55 DAP (D), and 55–60 (E). The GDR values on the x axis denote the boundary values for defining the GDR groups. The y axis on the left denotes the numbers of RILs related to the GDR groups. [file 12870_2020_2302_MOESM3_ESM.docx]

**
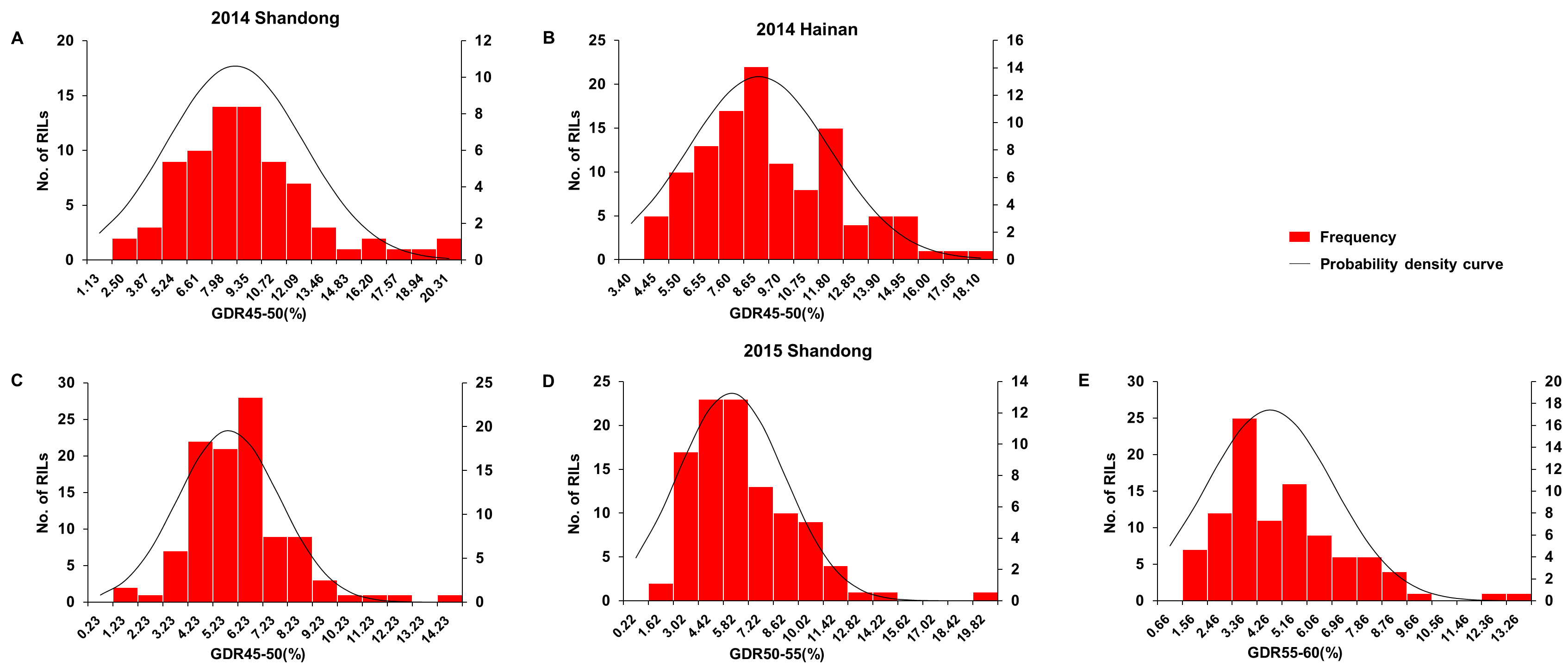
Figure S2** Histogram of the frequency distribution and probability density curve of GDR values for the RILs in the three field trials.

A-B, GDR values at 45–50 DAP in 2014 in Shandong (A), 2014 in Hainan (B). C-E, GDR values in 2015 in Shandong at 45–50 DAP (C), 50–55 DAP (D), and 55–60 (E). The GDR values on the *x* axis denote the boundary values for defining the GDR groups. The *y* axis on the left denotes the numbers of RILs related to the GDR groups.
