# Supplementary material for: Genetic dissection of grain water content and dehydration rate related to mechanical harvest in maize
Source: BMC Plant Biol. 2020 Mar 17;20:118. doi: 10.1186/s12870-020-2302-0 (PMC7076969; doi:10.1186/s12870-020-2302-0)
Supplement: Supplementary file 4 — Additional file 4: Table S2. Analysis of variance of GWC in three field trials. Sources: variation sources. df: degrees of freedom. SS: sum of squares. MS: mean squares. EMS: estimated mean square. P-value: significant difference among sources. *P < 0.05, **P < 0.01, ***P < 0.001. [file 12870_2020_2302_MOESM4_ESM.docx]

**Table S2** Analysis of variance of GWC in three field trials

| **Sources** | ***df*** | **SS** | **MS** | ***F-*value** | ***P-*value** | ***EMS*** | **Variance** |
| --- | --- | --- | --- | --- | --- | --- | --- |
| RILs | 128 | 1.73 | 0.01 | 11.77 | 1.46E-119 *** | $\text{σ}_{\text{e}}^{\text{2}}$*+*${\text{ }\text{rσ}}_{\text{G×E}}^{\text{2}}$*+* $\text{lrσ}_{\text{G}}^{\text{2}}$ | 1.25E-03 |
| Environments | 2 | 2.37 | 1.18 | 1032.27 | 7.27E-226 *** | $\text{σ}_{\text{e}}^{\text{2}}$ *+* $\text{rσ}_{\text{G×E}}^{\text{2}}\text{+ }\text{nr}\text{σ}_{\text{E}}^{\text{2}}$ | 4.56E-03 |
| Sample times | 1 | 1.52 | 1.52 | 1325.80 | 3.29E-174 *** | $\text{σ}_{\text{e}}^{\text{2}}$ *+*$\text{ }\text{nl}\text{σ}_{\text{S}}^{\text{2}}$ | 1.18E-02 |
| RILs× Environments | 185 | 0.47 | 2.52E-03 | 2.20 | 6.06E-14 *** | $\text{σ}_{\text{e}}^{\text{2}}\text{+}\text{rσ}_{\text{G×E}}^{\text{2}}$ | 6.85E-04 |
| Residuals | 830 | 0.95 | 1.15E-03 |  |  | $\text{σ}_{\text{e}}^{\text{2}}$ | 1.15E-03 |

**Sources**: variation sources.

***df***: degrees of freedom.

**SS**: sum of squares.

**MS**: mean squares.

***P*-value**: significant difference among sources. **P* < 0.05, ***P* < 0.01, ****P* < 0.001.

***EMS***: Estimated Mean Square.
