# Supplementary material for: Genetic dissection of grain water content and dehydration rate related to mechanical harvest in maize
Source: BMC Plant Biol. 2020 Mar 17;20:118. doi: 10.1186/s12870-020-2302-0 (PMC7076969; doi:10.1186/s12870-020-2302-0)
Supplement: Supplementary file 5 — Additional file 5: Table S3. Analysis of variance of GDR in three field trials. Sources: variation sources. df: degrees of freedom. SS: sum of squares. MS: mean squares. EMS: estimated mean square. P-value: significant difference among sources. *P < 0.05, **P < 0.01, ***P < 0.001. [file 12870_2020_2302_MOESM5_ESM.docx]

**Table S3** Analysis of variance of GDR in three field trials

| **Sources** | ***df*** | **SS** | **MS** | ***F-*value** | ***P-*value** | ***EMS*** | **Variance** |
| --- | --- | --- | --- | --- | --- | --- | --- |
| RILs | 127 | 0.29 | 2.32E-03 | 2.63 | 7.77E-11 *** | $\text{σ}_{\text{e}}^{\text{2}}$*+*${\text{ }\text{rσ}}_{\text{G×E}}^{\text{2}}$*+* $\text{lrσ}_{\text{G}}^{\text{2}}$ | 1.48E-04 |
| Environments | 2 | 0.12 | 0.06 | 67.16 | 8.92E-24 *** | $\text{σ}_{\text{e}}^{\text{2}}$ *+* $\text{rσ}_{\text{G×E}}^{\text{2}}\text{+ }\text{nr}\text{σ}_{\text{E}}^{\text{2}}$ | 2.27E-04 |
| Replications | 1 | 1.91E-03 | 1.91E-03 | 2.17 | 0.14 | $\text{σ}_{\text{e}}^{\text{2}}$ *+*$\text{ }\text{nl}\text{σ}_{\text{S}}^{\text{2}}$ | 7.98E-06 |
| RILs × Environments | 174 | 0.25 | 1.43E-03 | 1.62 | 2.89E-04 ** | $\text{σ}_{\text{e}}^{\text{2}}\text{+}\text{rσ}_{\text{G×E}}^{\text{2}}$ | 2.75E-04 |
| Residuals | 234 | 0.21 | 8.81E-04 |  |  | $\text{σ}_{\text{e}}^{\text{2}}$ | 8.81E-04 |

**Sources**: variation sources.

***df***: degrees of freedom.

**SS**: sum of squares.

**MS**: mean squares.

***P*-value**: significant difference among sources. **P* < 0.05, ***P* < 0.01, ****P* < 0.001.

***EMS***: Estimated Mean Square.
