# Supplementary material for: Genetic dissection of grain water content and dehydration rate related to mechanical harvest in maize
Source: BMC Plant Biol. 2020 Mar 17;20:118. doi: 10.1186/s12870-020-2302-0 (PMC7076969; doi:10.1186/s12870-020-2302-0)
Supplement: Supplementary file 6 — Additional file 6: Table S4. Correlation coefficient (r) for GWC and GDR between any two of three initial mapping trials. The correlation analysis was performed using the GWC at 45 and 50 DAP, as well as the GDR sampled at 45–50 DAP. GWC 45 DAP: GWC measured at 45 DAP. GWC 50 DAP: GWC measured at 50 DAP. GDR 45–50 DAP: GDR measured at 45–50 DAP. 14SD: The summer of 2014 in Shandong. 14HN: The winter of 2014 in Hainan. 15SD: The summer of 2015 in Shandong. The values in the table indicate the correlation coefficient (r) and its significant difference: *P < 0.05, **P < 0.01, ***P < 0.001. The correlation analysis was performed using the GWC at 45 and 50 DAP, as well as the GDR sampled at 45–50 DAP. [file 12870_2020_2302_MOESM6_ESM.docx]

**Table S4** Correlation coefficient (*r*) for GWC and GDR between any two of three initial mapping trials

| **GWC 45 DAP** | | | | **GWC 50 DAP** | | | | **GDR 45-50 DAP** | | | |
| --- | --- | --- | --- | --- | --- | --- | --- | --- | --- | --- | --- |
|  | 14SD | 14HN | 15SD |  |  |  |  |  |  |  |  |
| 14SD | 1 |  |  |  |  |  |  |  |  |  |  |
| 14HN | 0.34** | 1 |  |  |  |  |  |  |  |  |  |
| 15SD | 0.46*** | 0.42*** | 1 |  |  |  |  |  |  |  |  |
|  |  |  |  |  | 14SD | 14HN | 15SD |  |  |  |  |
|  |  |  |  | 14SD | 1 |  |  |  |  |  |  |
|  |  |  |  | 14HN | 0.46*** | 1 |  |  |  |  |  |
|  |  |  |  | 15SD | 0.35** | 0.43*** | 1 |  |  |  |  |
|  |  |  |  |  |  |  |  |  | 14SD | 14HN | 15SD |
|  |  |  |  |  |  |  |  | 14SD | 1 |  |  |
|  |  |  |  |  |  |  |  | 14HN | 0.34 * | 1 |  |
|  |  |  |  |  |  |  |  | 15SD | 0.18 | 0.18 | 1 |

**GWC 45 DAP**: GWC measured at 45 DAP.

**GWC 50 DAP**: GWC measured at 50 DAP.

**GDR 45-50 DAP**: GDR measured at 45-50 DAP.

14SD: The summer of 2014 in Shandong.

14HN: The winter of 2014 in Hainan.

15SD: The summer of 2015 in Shandong.

The values in the table indicate the correlation coefficient (*r*) and its significant difference: **P* < 0.05, ***P*< 0.01, ****P*<0.001.

The correlation analysis was performed using the GWC at 45 and 50 DAP, as well as the GDR sampled at 45-50 DAP.
