# Supplementary material for: Genetic dissection of grain water content and dehydration rate related to mechanical harvest in maize
Source: BMC Plant Biol. 2020 Mar 17;20:118. doi: 10.1186/s12870-020-2302-0 (PMC7076969; doi:10.1186/s12870-020-2302-0)
Supplement: Supplementary file 7 — Additional file 7: Table S5. Marker and genetic distance information for the 10 maize linkage groups. Chr.: chromosome; No. of Markers: number of markers on each chromosome. [file 12870_2020_2302_MOESM7_ESM.docx]

**Table S5** Marker and genetic distance information for the 10 maize linkage groups

| **Chr.** | **No. of Markers** | **Genetic distance (cM)** | **Average distance (cM)** |
| --- | --- | --- | --- |
| 1 | 93 | 247.82 | 2.66 |
| 2 | 64 | 92.59 | 1.00 |
| 3 | 91 | 221.75 | 2.38 |
| 4 | 76 | 149.76 | 1.61 |
| 5 | 81 | 143.91 | 1.55 |
| 6 | 86 | 116.61 | 1.25 |
| 7 | 56 | 114.24 | 1.23 |
| 8 | 103 | 127.11 | 1.37 |
| 9 | 65 | 185.37 | 1.99 |
| 10 | 67 | 123.32 | 1.33 |
| Total | 782 | 1522.48 | 1.95 |

Chr.: chromosome;

No. of Markers: number of markers on each chromosome.
