# Supplementary figures and images for: Genetic dissection of grain water content and dehydration rate related to mechanical harvest in maize
Source: BMC Plant Biol. 2020 Mar 17;20:118. doi: 10.1186/s12870-020-2302-0 (PMC7076969; doi:10.1186/s12870-020-2302-0)

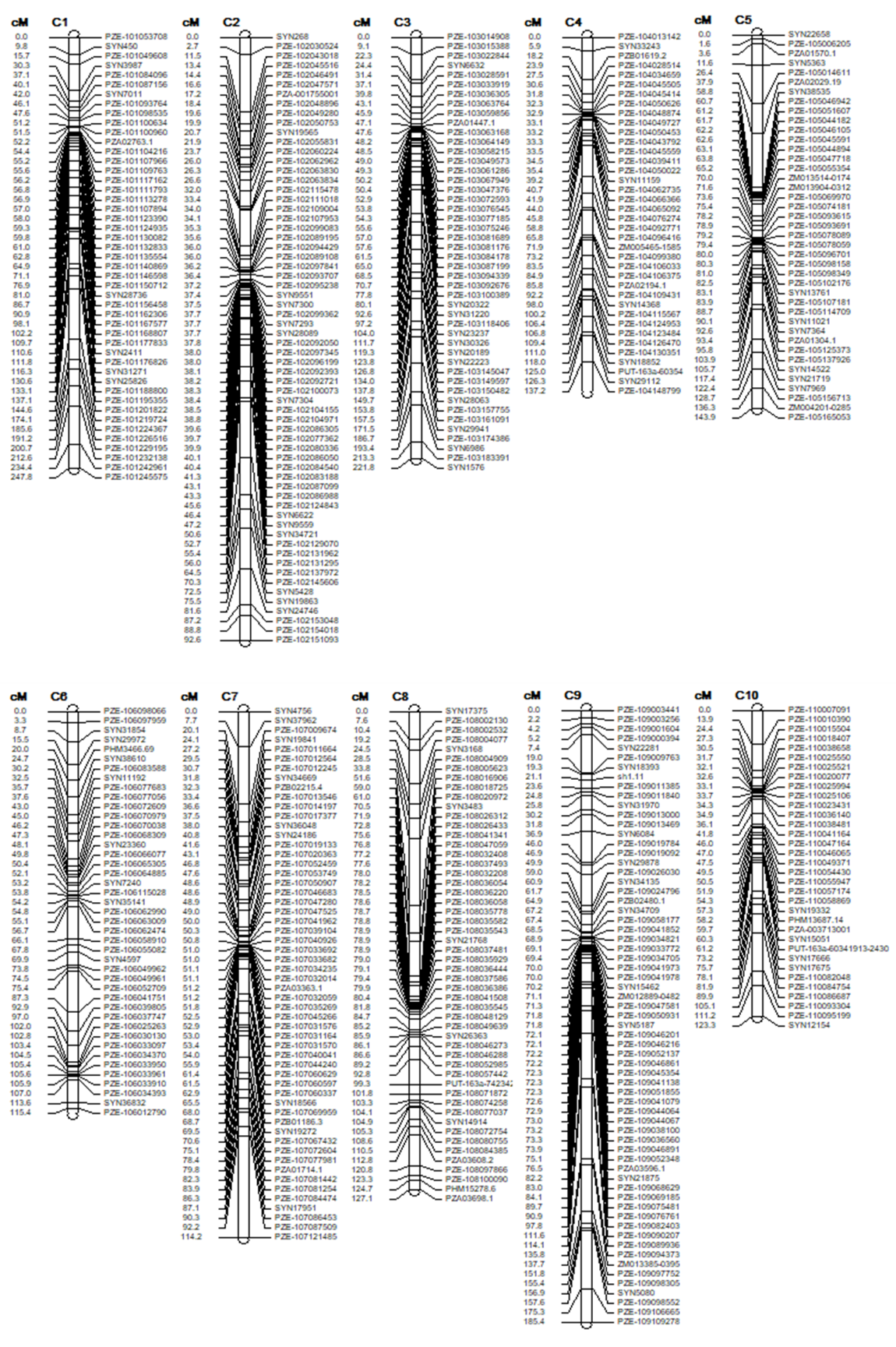
**Figure S3** Ten maize genetic linkage groups.

Supplement: Supplementary file 8 — Additional file 8: Figure S3. Ten maize genetic linkage groups. [file 12870_2020_2302_MOESM8_ESM.docx]

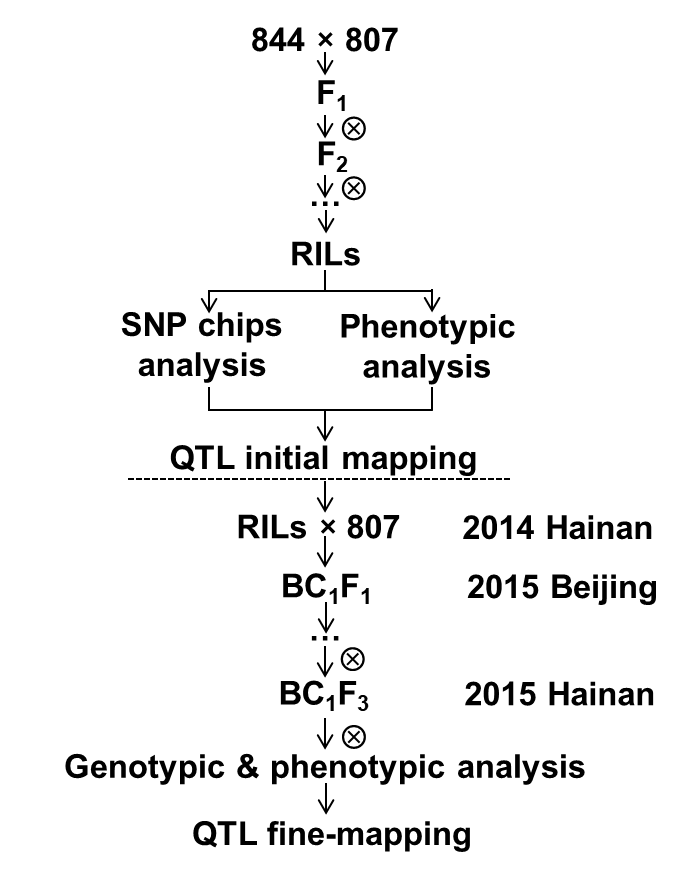
**Figure S7** Experimental flow chart for identifying QTL and fine-mapping.

Supplement: Supplementary file 18 — Additional file 18: Figure S7. Experimental flow chart for identifying QTL and fine-mapping. [file 12870_2020_2302_MOESM18_ESM.docx]
