# Supplementary material for: Genetic dissection of grain water content and dehydration rate related to mechanical harvest in maize
Source: BMC Plant Biol. 2020 Mar 17;20:118. doi: 10.1186/s12870-020-2302-0 (PMC7076969; doi:10.1186/s12870-020-2302-0)
Supplement: Supplementary file 9 — Additional file 9: Figure S4. Initial QTL mapping for GWC in three field trials. A, LOD profiles (upper) and additive genetic effects (lower) of ten maize chromosomes. B, QTL on chromosome 1. The legend with different lines and colors to the right indicates the sources of GWC. 1–6: GWC measured at 45 DAP with two replications (1–2) and their average value (3) and 50 DAP with two replications (4–5) and their average value (6) in Hainan in 2014. 7–12: GWC measured at 45 DAP with two replications (7–8) and their average value (9) and 50 DAP with two replications (10–11) and their average value (12) in Shandong in 2014. 13–24: GWC measured in Shandong in 2015, where 13–15 are two replications sampled at 45 DAP and their average value, 16–18 are two replications sampled at 50 DAP and their average value, 19–21 are two replications sampled at 55 DAP and their average value 22–24 are two replications sampled at 60 DAP and their average value. The x axes of both figures represent the genetic distance of different chromosomes. The y axis (upper) represents the LOD values for the QTL. The y axis (lower) represents the additive values for the QTL. [file 12870_2020_2302_MOESM9_ESM.docx]

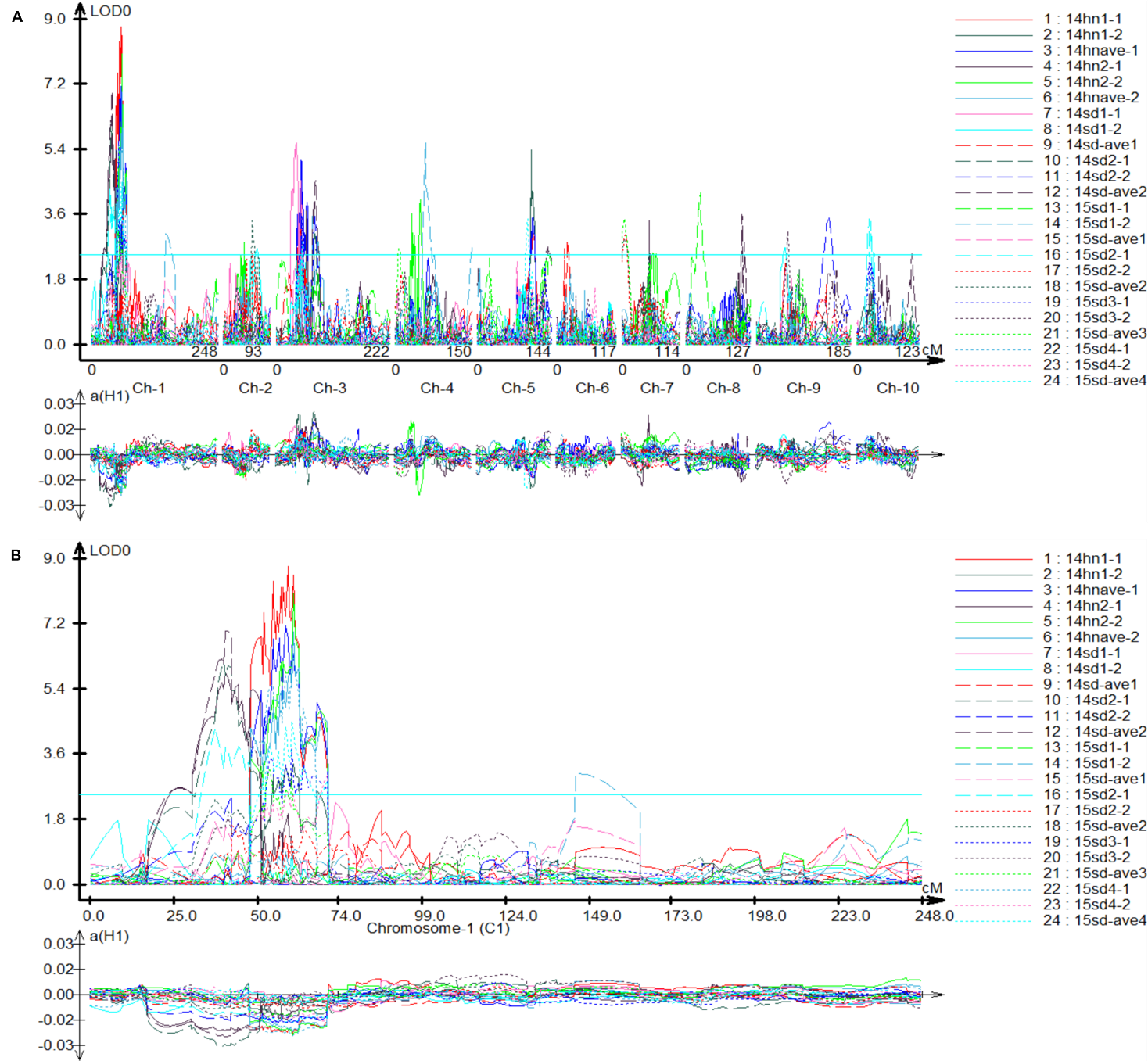


**Figure S4** Initial QTL mapping for GWC in three field trials.

A, LOD profiles (upper) and additive genetic effects (lower) of ten maize chromosomes. B, QTL on chromosome 1. The legend with different lines and colors to the right indicates the sources of GWC. 1-6: GWC measured at 45 DAP with two replications (1-2) and their average value (3) and 50 DAP with two replications (4-5) and their average value (6) in Hainan in 2014. 7-12: GWC measured at 45 DAP with two replications (7-8) and their average value (9) and 50 DAP with two replications (10-11) and their average value (12) in Shandong in 2014. 13-24: GWC measured in Shandong in 2015, where 13-15 are two replications sampled at 45 DAP and their average value, 16-18 are two replications sampled at 50 DAP and their average value, 19-21 are two replications sampled at 55 DAP and their average value 22-24 are two replications sampled at 60 DAP and their average value. The *x* axes of both figures represent the genetic distance of different chromosomes. The *y* axis (upper) represents the LOD values for the QTL. The *y* axis (lower) represents the additive values for the QTL.
