# Supplementary material for: Genetic dissection of grain water content and dehydration rate related to mechanical harvest in maize
Source: BMC Plant Biol. 2020 Mar 17;20:118. doi: 10.1186/s12870-020-2302-0 (PMC7076969; doi:10.1186/s12870-020-2302-0)
Supplement: Supplementary file 10 — Additional file 10: Figure S5. Initial QTL mapping results for GDR in three field trials. A, LOD profiles (upper) and additive genetic effects (lower) of ten maize chromosomes. B, QTL on chromosome 1. The legend with different lines and colors to the right indicates the sources of GDR. 1–2: GDR of two replications measured in Hainan in 2014. 3–4: GDR of two replications measured in Shandong in 2014. 5–7: GDR measured at 45–50 DAP, 50–55 DAP and 55–60 DAP from the first replication in Shandong in 2015. 8–10: GDR measured at 45–50 DAP, 50–55 DAP and 55–60 DAP from the second replication in Shandong in 2015. The x axes of both figures represent the genetic distance of different chromosomes. The y axis (upper) represents the LOD values for the QTL. The y axis (lower) represents the additive values for the QTL. [file 12870_2020_2302_MOESM10_ESM.docx]

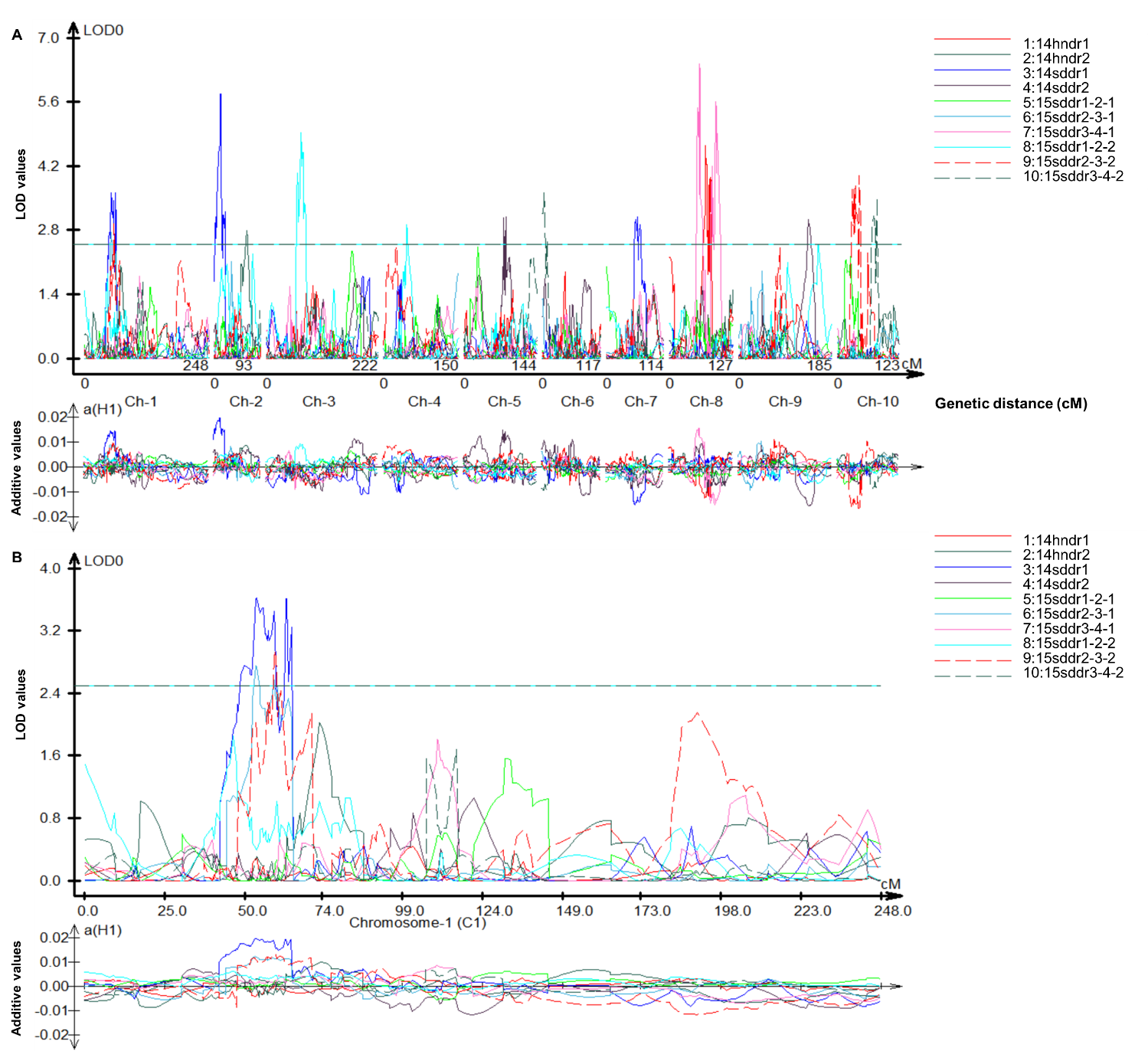


**Figure S5** Initial QTL mapping results for GDR in three field trials.

A, LOD profiles (upper) and additive genetic effects (lower) of ten maize chromosomes. B, QTL on chromosome 1. The legend with different lines and colors to the right indicates the sources of GDR. 1-2: GDR of two replications measured in Hainan in 2014. 3-4: GDR of two replications measured in Shandong in 2014. 5-7: GDR measured at 45-50 DAP, 50-55 DAP and 55-60 DAP from the first replication in Shandong in 2015. 8-10: GDR measured at 45-50 DAP, 50-55 DAP and 55-60 DAP from the second replication in Shandong in 2015. The *x* axes of both figures represent the genetic distance of different chromosomes. The *y* axis (upper) represents the LOD values for the QTL. The *y* axis (lower) represents the additive values for the QTL.
