# Supplementary material for: Genetic dissection of grain water content and dehydration rate related to mechanical harvest in maize
Source: BMC Plant Biol. 2020 Mar 17;20:118. doi: 10.1186/s12870-020-2302-0 (PMC7076969; doi:10.1186/s12870-020-2302-0)
Supplement: Supplementary file 11 — Additional file 11: Figure S6. Initial QTL mapping results for GWC and GDR from BLUP analysis. LOD profiles (upper) and additive genetic effects (lower) of ten maize chromosomes. The legend with different colors to the right indicates GWC at 45 and 50 DAP as well as GDR calculated by BLUP. The x axes of both figures represent the genetic distance of different chromosomes. The y axis (upper) represents the LOD values for the QTL. The y axis (lower) represents the additive values for the QTL. [file 12870_2020_2302_MOESM11_ESM.docx]

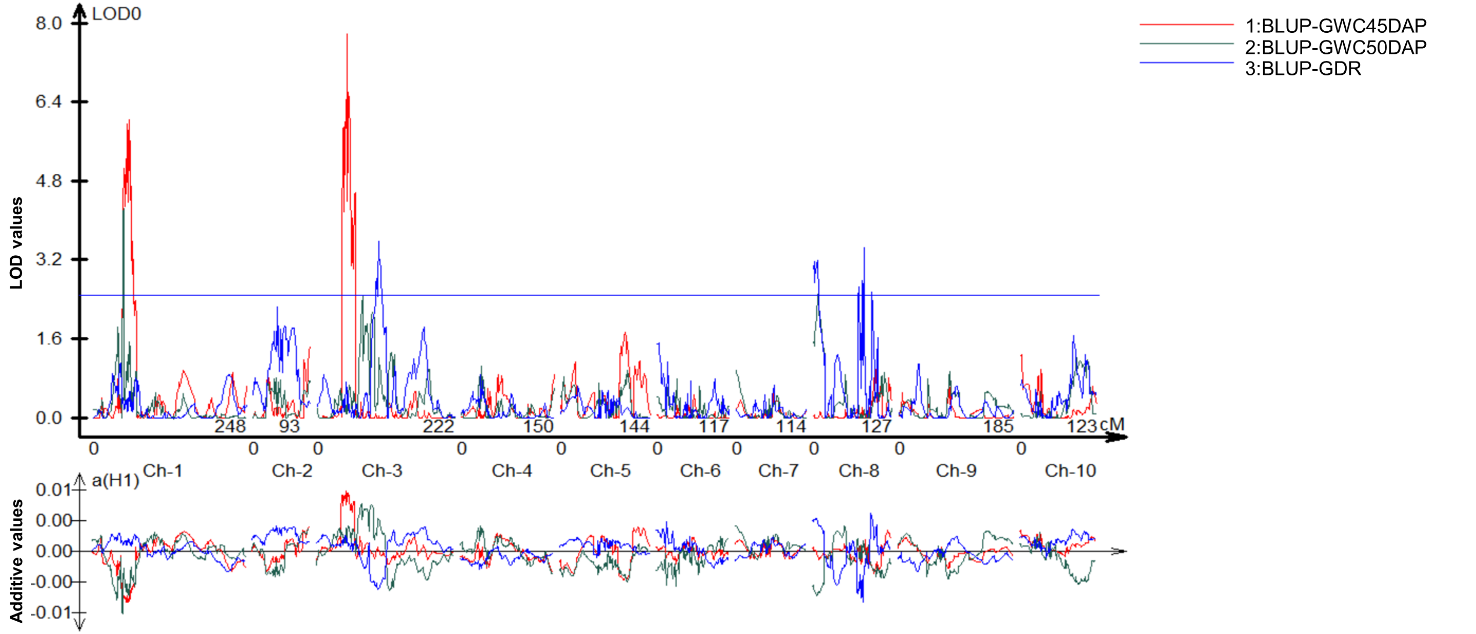


**Figure S6** Initial QTL mapping results for GWC and GDR from BLUP analysis.

LOD profiles (upper) and additive genetic effects (lower) of ten maize chromosomes. The legend with different colors to the right indicates GWC at 45 and 50 DAP as well as GDR calculated by BLUP. The *x* axes of both figures represent the genetic distance of different chromosomes. The *y* axis (upper) represents the LOD values for the QTL. The *y* axis (lower) represents the additive values for the QTL.
