# Supplementary material for: Genetic dissection of grain water content and dehydration rate related to mechanical harvest in maize
Source: BMC Plant Biol. 2020 Mar 17;20:118. doi: 10.1186/s12870-020-2302-0 (PMC7076969; doi:10.1186/s12870-020-2302-0)
Supplement: Supplementary file 12 — Additional file 12: Table S6. Initial QTL mapping of GWC and GDR in the summer of 2014 in Shandong. Traits: the phenotypes. QTL: the names of QTL which were detected in the initial QTL mapping. Sources: the sources of phenotypes, 14sd1–1 and 14sd1–2 represent the phenotypes sampled at 45 DAP of replicate 1 and replicate 2 in the summer of 2014 in Shandong, respectively; 14sd2–1 and 14sd2–2 represent the phenotypes sampled at 50 DAP of replicate 1 and replicate 2 in the summer of 2014 in Shandong, respectively; 14sd-ave1 and 14sd-ave2 represent the average phenotypes sampled at 45 and 50 DAP, respectively. 14sddr1 and 14sddr2 represent the GDR between 45 and 50 DAP of replicate 1 and replicate 2, respectively. Rep: the names of replications related to phenotypic data. “AVE”: represents the phenotypes from the average value of R1 and R2. DAP: Days after pollination. Bins: the location of the QTL in the chromosomes. Flanking SNPs: the SNPs at the both sides of QTL. Physical Location (Mb): the physical location of the QTL. CI (Mb): size of confident interval. AE: additive effect. R2: explained phenotypic variation. [file 12870_2020_2302_MOESM12_ESM.docx]

**Table S6** Initial QTL mapping of GWC and GDR in the summer of 2014 in Shandong

| **Traits** | **QTL** | **Sources** | **Rep** | **DAP** | **Bins** | **Flanking SNPs** | **Physical Location (Mb)** | **CI (Mb)** | **LOD** | **AE (%)** | ***R*^2^** |
| --- | --- | --- | --- | --- | --- | --- | --- | --- | --- | --- | --- |
| GWC | *qGwc2.1* | 14sd1-1 | R1 | 45 | 2.02-2.03 | PZE-102030524-PZE-102046491 | 14.27-23.88 | 9.61 | 3.68 | 1.79 | 12.92% |
|  | *qGwc3.2* | 14sd1-1 | R1 | 45 | 3.04 | PZE-103026528-PZE-103061612 | 19.65-106.24 | 86.59 | 4.37 | 1.57 | 15.74% |
|  | *qGwc2.3* | 14sd1-2 | R2 | 45 | 2.06-2.07 | PZE-102131962-SYN5428 | 182.34-190.16 | 7.82 | 2.85 | 1.33 | 10.19% |
|  | *qGwc3.2* | 14sd1-2 | R2 | 45 | 3.04-3.05 | PZE-103036305-PZE-103084178 | 29.8-139.51 | 109.71 | 2.53 | 1.34 | 8.54% |
|  | *qGwc9.1* | 14sd1-2 | R2 | 45 | 9.02-9.03 | PZE-109019784-SYN34709 | 20.23-99.41 | 79.18 | 2.55 | 1.28 | 10.17% |
|  | *qGwc3.1* | 14sd-ave1 | AVE | 45 | 3.02-3.04 | PZE-103015388-PZE-103026528 | 8.27-19.65 | 11.38 | 2.67 | 1.34 | 11.43% |
|  | *qGwc6.2* | 14sd-ave1 | AVE | 45 | 6.08 | SYN24806-PZE-106080884 | 167.03-137.83 | 29.20 | 2.97 | 1.10 | 9.19% |
|  | *qGwc1.1* | 14sd2-1 | R1 | 50 | 1.04-1.05 | SYN3987-PZE-101101518 | 65.66-98.76 | 33.10 | 5.70 | -2.68 | 16.23% |
|  | *qGwc3.2* | 14sd2-1 | R1 | 50 | 3.04 | PZE-103036305-PZE-103054563 | 29.8-63.69 | 33.89 | 3.13 | 3.90 | 15.55% |
|  | *qGwc3.3* | 14sd2-1 | R1 | 50 | 3.05-3.06 | PZE-103094339-PZE-103110355 | 155.32-170.68 | 15.36 | 2.93 | 2.98 | 11.29% |
|  | *qGwc1.1* | 14sd2-2 | R2 | 50 | 1.04-1.05 | SYN3987-PZE-101101518 | 65.66-98.76 | 33.10 | 2.78 | -1.77 | 10.32% |
|  | *qGwc3.3* | 14sd2-2 | R2 | 50 | 3.05-3.06 | PZE-103094339-PZE-103110355 | 155.32-170.68 | 15.36 | 3.01 | 1.91 | 11.63% |
|  | *qGwc9.3* | 14sd2-2 | R2 | 50 | 9.06 | PZE-109089936-PZE-109097752 | 137.79-142.95 | 5.16 | 3.45 | 1.92 | 13.06% |
|  | *qGwc1.1* | 14sd-ave2 | AVE | 50 | 1.04-1.05 | SYN3987-PZE-101101518 | 65.66-98.76 | 33.10 | 6.53 | -2.65 | 24.47% |
|  | *qGwc3.3* | 14sd-ave2 | AVE | 50 | 3.05-3.06 | PZE-103087199-PZE-103110355 | 144.69-170.68 | 25.99 | 4.42 | 2.18 | 14.86% |
|  | *qGwc8.6* | 14sd-ave2 | AVE | 50 | 8.05-8.06 | PZE-108080755-PZE-108097866 | 137.22-153.8 | 16.58 | 3.01 | -1.61 | 9.56% |
|  | *qGwc10.4* | 14sd-ave2 | AVE | 50 | 10.06 | SYN37373-PZE-110096735 | 140-142.85 | 2.85 | 2.72 | -1.62 | 9.40% |
| GDR | *qGdr1.2* | 14sddr1 | R1 | 45-50 | 1.05 | PZE-101093040-PZE-101135767 | 85.72-175.64 | 89.92 | 3.47 | 1.64 | 14.46% |
|  | *qGdr2.1* | 14sddr1 | R1 | 45-45 | 2.03-2.04 | SYN268-PZE-102055831 | 16.12-33.59 | 17.47 | 5.81 | 2.27 | 26.12% |
|  | *qGdr7.2* | 14sddr1 | R1 | 45-50 | 7.02-7.03 | PZE-107045266-PZE-107077981 | 92.39-133.03 | 40.64 | 3.17 | -1.57 | 12.94% |
|  | *qGdr5.1* | 14sddr2 | R2 | 45-50 | 5.04-5.05 | PZE-105069970-SYN7361 | 74.34-176.12 | 101.78 | 2.93 | 1.56 | 10.16% |
|  | *qGdr9.3* | 14sddr2 | R2 | 45-50 | 9.06 | PZE-109089936-PZE-109097752 | 137.79-142.95 | 5.16 | 3.95 | -1.91 | 15.01% |

**Traits**: the phenotypes. **QTL**: the names of QTL which were detected in the initial QTL mapping.

**Sources**: the sources of phenotypes, 14sd1-1 and 14sd1-2 represent the phenotypes sampled at 45 DAP of replicate 1 and replicate 2 in the summer of 2014 in Shandong, respectively; 14sd2-1 and 14sd2-2 represent the phenotypes sampled at 50 DAP of replicate 1 and replicate 2 in the summer of 2014 in Shandong, respectively; 14sd-ave1 and 14sd-ave2 represent the average phenotypes sampled at 45 and 50 DAP, respectively. 14sddr1 and 14sddr2 represent the GDR between 45–50 DAP of replicate 1 and replicate 2, respectively.

**Rep**: the names of replications related to phenotypic data. “AVE”: represents the phenotypes from the average value of R1 and R2.

**DAP**: Days after pollination.

**Bins**: the location of the QTL in the chromosomes.

**Flanking SNPs**: the SNPs at the both sides of QTL.

**Physical Location (Mb)**: the physical location of the QTL.

**CI (Mb)**: size of confident interval.

**AE**: additive effect.

***R^2^***: explained phenotypic variation.
