# Supplementary material for: Genetic dissection of grain water content and dehydration rate related to mechanical harvest in maize
Source: BMC Plant Biol. 2020 Mar 17;20:118. doi: 10.1186/s12870-020-2302-0 (PMC7076969; doi:10.1186/s12870-020-2302-0)
Supplement: Supplementary file 13 — Additional file 13: Table S7. Initial QTL mapping of GWC and GDR in the winter of 2014 in Hainan. Traits: the phenotypes. QTL: the names of QTL which were detected in the initial QTL mapping. Sources: the sources of phenotypes, 14hn1–1 and 14hn1–2 represent the phenotypes sampled at 45 DAP of replicate 1 and replicate 2 in the winter of 2014 in Hainan, respectively; 14hn2–1 and 14hn2–2 represent the phenotypes sampled at 50 DAP of replicate 1 and replicate 2 in the winter of 2014 in Hainan, respectively; 14hn-ave1 and 14hn-ave2 represent the average phenotypes sampled at 45 and 50 DAP, respectively. 14hndr1 and 14hndr2 represent the GDR between 45 and 50 DAP of replicate 1 and replicate 2, respectively. Rep: the names of replications related to phenotypic data. “AVE”: represents the phenotypes from the average value of R1 and R2. DAP: Days after pollination. Bins: the location of the QTL in the chromosomes. Flanking SNPs: the SNPs at the both sides of QTL. Physical Location (Mb): the physical location of the QTL. CI (Mb): size of confident interval. AE: additive effect. R2: explained phenotypic variation. [file 12870_2020_2302_MOESM13_ESM.docx]

**Table S7** Initial QTL mapping of GWC and GDR in the winter of 2014 in Hainan

| **Traits** | **QTL** | **Sources** | **Rep** | **DAP** | **Bins** | **Flanking SNPs** | **Physical Location (Mb)** | **CI (Mb)** | **LOD** | **AE (%)** | ***R*^2^** |
| --- | --- | --- | --- | --- | --- | --- | --- | --- | --- | --- | --- |
| GWC | *qGwc1.2* | 14hn1-1 | R1 | 45 | 1.05-1.06 | PZA00944.1-PZE-101146598 | 89.00-189.77 | 100.77 | 8.34 | -2.32 | 23.74% |
|  | *qGwc3.2* | 14hn1-1 | R1 | 45 | 3.04-3.05 | PZE-103033919-PZE-103082105 | 26.45-136.08 | 109.63 | 3.81 | 1.45 | 9.77% |
|  | *qGwc1.2* | 14hn1-2 | R2 | 45 | 1.05-1.06 | PZA00944.1-PZE-101146598 | 89.00-189.77 | 100.77 | 3.07 | -1.26 | 7.66% |
|  | *qGwc3.2* | 14hn1-2 | R2 | 45 | 3.04-3.05 | PZE-103033919-PZE-103082105 | 26.45-136.08 | 109.63 | 2.74 | 1.28 | 7.30% |
|  | *qGwc5.2* | 14hn1-2 | R2 | 45 | 5.05-5.06 | SYN20663-SYN14995 | 181.89-203.82 | 21.93 | 4.90 | -2.06 | 13.97% |
|  | *qGwc1.2* | 14hnave-1 | AVE | 45 | 1.05-1.06 | PZA00944.1-PZE-101146598 | 89.00-189.77 | 100.77 | 6.65 | -1.74 | 17.50% |
|  | *qGwc3.2* | 14hnave-1 | AVE | 45 | 3.04-3.05 | PZE-103033919-PZE-103084178 | 26.45-139.51 | 113.06 | 5.30 | 1.60 | 13.39% |
|  | *qGwc5.2* | 14hnave-1 | AVE | 45 | 5.05-5.06 | SYN20663-SYN14995 | 181.89-203.82 | 21.93 | 2.92 | -1.22 | 8.33% |
|  | *qGwc1.1* | 14hn2-1 | R1 | 50 | 1.04-1.05 | SYN3987-PZE-101109414 | 65.66-117.16 | 51.50 | 5.68 | -2.32 | 17.55% |
|  | *qGwc3.2* | 14hn2-1 | R1 | 50 | 3.04-3.05 | PZE-103061612-PZE-103084178 | 106.24-139.51 | 33.27 | 3.69 | 1.90 | 10.31% |
|  | *qGwc7.3* | 14hn2-1 | R1 | 50 | 7.02 | PZE-107034235-PZE-107044240 | 47.4-88.12 | 40.72 | 3.93 | 3.22 | 10.95% |
|  | *qGwc1.2* | 14hn2-2 | R2 | 50 | 1.05-1.06 | PZA00944.1-PZE-101146598 | 89-189.77 | 100.77 | 7.81 | -2.51 | 22.21% |
|  | *qGwc2.2* | 14hn2-2 | R2 | 50 | 2.05-2.06 | SYN7304-SYN9559 | 110.82-175.83 | 65.01 | 2.75 | -1.39 | 6.88% |
|  | *qGwc4.1* | 14hn2-2 | R2 | 50 | 4.05-4.06 | PZE-104062792-PZE-104092771 | 124.28-168.83 | 44.55 | 5.08 | -2.81 | 13.59% |
|  | *qGwc1.2* | 14hnave-2 | AVE | 50 | 1.05-1.06 | PZA00944.1-PZE-101146598 | 89-189.77 | 100.77 | 6.98 | -2.34 | 19.57% |
|  | *qGwc3.3* | 14hnave-2 | AVE | 50 | 3.05-3.06 | PZE-103094339-SYN31220 | 155.32-180.98 | 25.66 | 2.76 | 1.49 | 7.31% |
| GDR | *qGdr8.4* | 14hndr1 | R1 | 45-50 | 8.02-8.03 | PZE-108020972-PZE-108047536 | 19.86-79.93 | 60.07 | 4.77 | -1.37 | 14.94% |
|  | *qGdr2.3* | 14hndr2 | R2 | 45-50 | 2.06-2.07 | PZE-102129070-SYN19863 | 178.98-191.15 | 12.17 | 3.10 | 1.06 | 10.05% |

**Traits**: the phenotypes. **QTL**: the names of QTL which were detected in the initial QTL mapping.

**Sources**: the sources of phenotypes, 14hn1-1 and 14hn1-2 represent the phenotypes sampled at 45 DAP of replicate 1 and replicate 2 in the winter of 2014 in Hainan, respectively; 14hn2-1 and 14hn2-2 represent the phenotypes sampled at 50 DAP of replicate 1 and replicate 2 in the winter of 2014 in Hainan, respectively; 14hn-ave1 and 14hn-ave2 represent the average phenotypes sampled at 45 and 50 DAP, respectively. 14hndr1 and 14hndr2 represent the GDR between 45–50 DAP of replicate 1 and replicate 2, respectively.

**Rep**: the names of replications related to phenotypic data. “AVE”: represents the phenotypes from the average value of R1 and R2.

**DAP**: Days after pollination.

**Bins**: the location of the QTL in the chromosomes.

**Flanking SNPs**: the SNPs at the both sides of QTL.

**Physical Location (Mb)**: the physical location of the QTL.

**CI (Mb)**: size of confident interval.

**AE**: additive effect.

***R^2^***: explained phenotypic variation.
