# Supplementary material for: Genetic dissection of grain water content and dehydration rate related to mechanical harvest in maize
Source: BMC Plant Biol. 2020 Mar 17;20:118. doi: 10.1186/s12870-020-2302-0 (PMC7076969; doi:10.1186/s12870-020-2302-0)
Supplement: Supplementary file 14 — Additional file 14: Table S8. Initial QTL mapping of GWC and GDR in the summer of 2015 in Shandong. Traits: the phenotypes. QTL: the names of QTL which were detected in the initial QTL mapping. Sources: the sources of phenotypes, 15sd1–1 and 15sd1–2 represent the phenotypes sampled at 45 DAP of replicate 1 and replicate 2 in the summer of 2015 in Shandong, respectively; 15sd2–1 and 15sd2–2 represent the phenotypes sampled at 50 DAP of replicate 1 and replicate 2 in the summer of 2015 in Shandong, respectively; 15sd3–1 and 15sd3–2 represent the phenotypes sampled at 55 DAP of replicate 1 and replicate 2 in the summer of 2015 in Shandong, respectively; 15sd4–1 and 15sd4–2 represent the phenotypes sampled at 60 DAP of replicate 1 and replicate 2 in the summer of 2015 in Shandong, respectively; 15sd-ave1, 15sd-ave2, 15sd-ave3 and 15sd-ave4 represent the average phenotypes sampled at 45, 50, 55 and 60 DAP, respectively. 15sddr1–2-1, 15sddr1–2-2 represent the GDR between 45 and 50 DAP of replicate 1 and replicate 2, respectively. 15sddr2–3-1, 15sddr2–3-2 represent the GDR between 50 and 55 DAP of replicate 1 and replicate 2, respectively. 15sddr3–4-1, 15sddr3–4-2 represent the GDR between 55 and 60 DAP of replicate 1 and replicate 2, respectively. Rep: the names of replications related to phenotypic data. “AVE”: represents the phenotypes from the average value of R1 and R2. DAP: Days after pollination. Bins: the location of the QTL in the chromosomes. Flanking SNPs: the SNPs at the both sides of QTL. Physical Location (Mb): the physical location of the QTL. CI (Mb): size of confident interval. AE: additive effect. R2: explained phenotypic variation. [file 12870_2020_2302_MOESM14_ESM.docx]

**Table S8** Initial QTL mapping of GWC and GDR in the summer of 2015 in Shandong

| **Traits** | **QTL** | **Sources** | **Rep** | **DAP** | **Bins** | **Flanking SNPs** | **Physical Location (Mb)** | **CI (Mb)** | **LOD** | **AE (%)** | ***R*^2^** |
| --- | --- | --- | --- | --- | --- | --- | --- | --- | --- | --- | --- |
| GWC | *qGwc3.1* | 15sd1-1 | R1 | 45 | 3.02-3.04 | PZE-103014908-PZE-103022844 | 8.04-15.06 | 7.02 | 3.00 | 1.07 | 13.05% |
|  | *qGwc8.2* | 15sd1-1 | R1 | 45 | 8.01 | PZE-108004077-PZE-108005623 | 4.18-5.68 | 1.5 | 5.30 | -1.29 | 19.47% |
|  | *qGwc4.2* | 15sd1-2 | R2 | 45 | 4.06-4.07 | SYN13970-PZE-104102768 | 153.63-178.89 | 25.26 | 4.30 | 1.25 | 14.44% |
|  | *qGwc3.2* | 15sd-ave1 | AVE | 45 | 3.04-3.05 | PZE-103036305-PZE-103082105 | 29.8-136.08 | 106.28 | 2.97 | 0.87 | 9.33% |
|  | *qGwc1.1* | 15sd2-1 | R1 | 50 | 1.04-1.05 | SYN3987-PZA00944.1 | 65.66-89.00 | 23.34 | 3.93 | -1.19 | 12.81% |
|  | *qGwc3.2* | 15sd2-1 | R1 | 50 | 3.04 | PZE-103026528-PZE-103061612 | 19.65-106.24 | 86.59 | 2.88 | 1.04 | 9.10% |
|  | *qGwc10.1* | 15sd2-1 | R1 | 50 | 10.02-10.03 | PZE-110010390-PZE-110020162 | 8.93-25.56 | 16.63 | 3.56 | 1.09 | 11.40% |
|  | *qGwc2.3* | 15sd2-2 | R2 | 50 | 2.06-2.07 | PZE-102131962-PZE-102145606 | 182.34-192.58 | 10.24 | 4.40 | 2.21 | 14.95% |
|  | *qGwc7.1* | 15sd2-2 | R2 | 50 | 7.01 | SYN4756-PZE-107009674 | 5.15-6.3 | 1.15 | 2.94 | 1.18 | 9.84% |
|  | *qGwc2.3* | 15sd-ave2 | AVE | 50 | 2.06 | SYN34721-PZE-102137972 | 177.75-186.79 | 9.04 | 4.45 | 1.50 | 14.02% |
|  | *qGwc7.1* | 15sd-ave2 | AVE | 50 | 7.01 | SYN4756-PZE-107009674 | 5.15-6.3 | 1.15 | 3.21 | 1.08 | 10.00% |
|  | *qGwc1.2* | 15sd3-1 | R1 | 55 | 1.05-1.06 | PZA00944.1-PZE-101146598 | 89-189.77 | 100.77 | 3.72 | -1.59 | 11.97% |
|  | *qGwc3.2* | 15sd3-1 | R1 | 55 | 3.04-3.05 | PZE-103033919-PZE-103082105 | 26.45-136.08 | 109.63 | 2.67 | 1.37 | 8.54% |
|  | *qGwc5.3* | 15sd3-1 | R1 | 55 | 5.07 | PZE-105156153-ZM004201-0285 | 205.38-207.72 | 2.34 | 2.68 | 1.47 | 8.80% |
|  | *qGwc10.1* | 15sd3-1 | R1 | 55 | 10.02-10.03 | PZE-110013181-PZE-110038481 | 11.81-73.62 | 61.81 | 2.64 | 1.27 | 8.19% |
|  | *qGwc9.2* | 15sd3-2 | R2 | 55 | 9.02-9.03 | SYN29878-PZE-109044067 | 20.82-74.59 | 53.77 | 2.89 | -1.81 | 11.01% |
|  | *qGwc1.2* | 15sd-ave3 | AVE | 55 | 1.05-1.06 | PZA00944.1-PZE-101146598 | 89-189.77 | 100.77 | 2.68 | -1.30 | 7.99% |
|  | *qGwc7.1* | 15sd-ave3 | AVE | 55 | 7.01 | SYN4756-PZE-107009674 | 5.15-6.3 | 1.15 | 2.62 | 1.39 | 9.26% |
|  | *qGwc1.2* | 15sd4-1 | R1 | 60 | 1.05-1.06 | PZA00944.1-PZE-101146598 | 89-189.77 | 100.77 | 6.81 | -2.63 | 24.78% |
|  | *qGwc1.2* | 15sd4-2 | R2 | 60 | 1.05-1.06 | PZA00944.1-PZE-101146598 | 89-189.77 | 100.77 | 3.03 | -1.64 | 10.86% |
|  | *qGwc1.2* | 15sd-ave4 | AVE | 60 | 1.05-1.06 | PZA00944.1-PZE-101146598 | 89-189.77 | 100.77 | 4.75 | -1.81 | 13.99% |
|  | *qGwc3.2* | 15sd-ave4 | AVE | 60 | 3.04-3.05 | PZE-103036305-PZE-103084178 | 29.8-139.51 | 109.71 | 2.68 | 1.42 | 6.92% |
|  | *qGwc5.2* | 15sd-ave4 | AVE | 60 | 5.05 | SYN7361-PZE-105137926 | 176.12-192.86 | 16.74 | 3.48 | -2.00 | 10.23% |
| GDR | *qGdr1.2* | 15sddr2-3-1 | R1 | 50-55 | 1.05 | PZE-101093040-PZE-101135767 | 85.72-175.64 | 89.92 | 2.94 | 1.09 | 11.38% |
|  | *qGdr8.3* | 15sddr3-4-1 | R1 | 55-60 | 8.02-8.03 | PZE-108016906-SYN3483 | 16.75-22.68 | 5.94 | 5.68 | 1.83 | 28.54% |
|  | *qGdr8.5* | 15sddr3-4-1 | R1 | 55-60 | 8.03-8.04 | PZE-108035545-PZE-108074338 | 52.21-129.62 | 77.42 | 5.46 | -1.56 | 23.54% |
|  | *qGdr3.3* | 15sddr1-2-2 | R2 | 45-50 | 3.05 | PZE-103082105-PZE-103100389 | 136.08-160.67 | 24.58 | 4.30 | 0.98 | 15.23% |
|  | *qGdr9.4* | 15sddr1-2-2 | R2 | 45-50 | 9.04-9.06 | PZE-109075481-PZE-109090207 | 122.31-138.14 | 15.84 | 2.64 | 0.72 | 8.75% |
|  | *qGdr9.5* | 15sddr1-2-2 | R2 | 45-50 | 9.06-9.07 | ZM013385-0395-PZE-109106665 | 140.77-148.45 | 7.67 | 3.12 | -0.78 | 10.15% |
|  | *qGdr1.2* | 15sddr2-3-2 | R2 | 50-55 | 1.05-1.06 | PZA00944.1-PZE-101146598 | 89-189.77 | 100.77 | 2.95 | 1.16 | 9.44% |
|  | *qGdr10.1* | 15sddr2-3-2 | R2 | 50-55 | 10.02-10.03 | PZE-110013181-PZE-110047164 | 11.81-88.4 | 76.59 | 4.93 | -1.95 | 16.41% |
|  | *qGdr10.2* | 15sddr2-3-2 | R2 | 50-55 | 10.04-10.05 | PZE-110062675-PZE-110079903 | 117.75-134.22 | 16.47 | 2.53 | 1.37 | 8.10% |
|  | *qGdr6.1* | 15sddr3-4-2 | R2 | 55-60 | 6.05-6.07 | PZE-106098066-SYN35815 | 151.87-167.98 | 16.11 | 3.17 | -1.02 | 13.11% |
|  | *qGdr10.3* | 15sddr3-4-2 | R2 | 55-60 | 10.05-10.06 | PZE-110073847-PZE-110086687 | 130.31-137.75 | 7.44 | 3.44 | -1.00 | 15.99% |

**Traits**: the phenotypes. **QTL**: the names of QTL which were detected in the initial QTL mapping.

**Sources**: the sources of phenotypes, 15sd1-1 and 15sd1-2 represent the phenotypes sampled at 45 DAP of replicate 1 and replicate 2 in the summer of 2015 in Shandong, respectively; 15sd2-1 and 15sd2-2 represent the phenotypes sampled at 50 DAP of replicate 1 and replicate 2 in the summer of 2015 in Shandong, respectively; 15sd3-1 and 15sd3-2 represent the phenotypes sampled at 55 DAP of replicate 1 and replicate 2 in the summer of 2015 in Shandong, respectively; 15sd4-1 and 15sd4-2 represent the phenotypes sampled at 60 DAP of replicate 1 and replicate 2 in the summer of 2015 in Shandong, respectively; 15sd-ave1, 15sd-ave2, 15sd-ave3 and 15sd-ave4 represent the average phenotypes sampled at 45, 50, 55 and 60 DAP, respectively. 15sddr1-2-1, 15sddr1-2-2 represent the GDR between 45–50 DAP of replicate 1 and replicate 2, respectively. 15sddr2-3-1, 15sddr2-3-2 represent the GDR between 50–55 DAP of replicate 1 and replicate 2, respectively. 15sddr3-4-1, 15sddr3-4-2 represent the GDR between 55–60 DAP of replicate 1 and replicate 2, respectively.

**Rep**: the names of replications related to phenotypic data. “AVE”: represents the phenotypes from the average value of R1 and R2.

**DAP**: Days after pollination.

**Bins**: the location of the QTL in the chromosomes.

**Flanking SNPs**: the SNPs at the both sides of QTL.

**Physical Location (Mb)**: the physical location of the QTL.

**CI (Mb)**: size of confident interval.

**AE**: additive effect.

***R^2^***: explained phenotypic variation.
