# Supplementary material for: Genetic dissection of grain water content and dehydration rate related to mechanical harvest in maize
Source: BMC Plant Biol. 2020 Mar 17;20:118. doi: 10.1186/s12870-020-2302-0 (PMC7076969; doi:10.1186/s12870-020-2302-0)
Supplement: Supplementary file 15 — Additional file 15: Table S9. Initial QTL mappings of GWC and GDR from BLUP analysis in three field trials. DAP: Days after pollination. Bins: the location of the QTL in the chromosomes. Flanking SNPs: the SNPs at the both sides of QTL. Physical Location (Mb): the physical location of the QTL. CI (Mb): size of confident interval. AE: additive effect. R2: explained phenotypic variation. [file 12870_2020_2302_MOESM15_ESM.docx]

**Table S9** Initial QTL mappings of GWC and GDR from BLUP analysis in three field trials

| **QTL** | **DAP** | **Bins** | **Flanking SNPs** | **Physical Location (Mb)** | **CI (Mb)** | **LOD** | **AE (%)** | ***R^2^*** |
| --- | --- | --- | --- | --- | --- | --- | --- | --- |
| *qBGwc1.2* | 45 | 1.05-1.06 | PZA00944.1-PZE-101146598 | 89.00-189.77 | 100.77 | 4.23 | -0.68 | 14.01% |
| *qBGwc3.2* | 45 | 3.04-3.05 | PZE-103036305-PZE-103084178 | 29.80-139.51 | 109.71 | 7.79 | 0.83 | 19.06% |
| *qBGwc1.2* | 50 | 1.05 | PZA00944.1-PZE-101101518 | 89.00-98.76 | 9.76 | 5.88 | -0.84 | 12.40% |
| *qBGwc3.3* | 50 | 3.06 | PZE-103110355-SYN31220 | 170.68-180.98 | 10.30 | 2.82 | 0.71 | 8.67% |
| *qBGwc8.1* | 50 | 8.00-8.01 | SYN17375-SYN16886 | 0.40-3.43 | 3.03 | 3.10 | -0.66 | 7.30% |
| *qBGdr3.3* | 45-50 | 3.06 | PZE-103110355-SYN30326 | 170.68-187.21 | 16.53 | 3.64 | -0.51 | 9.74% |
| *qBGdr8.1* | 45-50 | 8.00-8.01 | SYN17375-SYN16886 | 0.40-3.43 | 3.03 | 3.06 | 0.45 | 7.58% |
| *qBGdr8.4* | 45-50 | 8.03 | SYN3483-PZE-108047536 | 22.68-79.93 | 57.25 | 4.00 | -0.63 | 10.55% |

**DAP**: Days after pollination.

**Bins**: the location of the QTL in the chromosomes.

**Flanking SNPs**: the SNPs at the both sides of QTL.

**Physical Location (Mb)**: the physical location of the QTL.

**CI (Mb)**: size of confident interval.

**AE**: additive effect.

***R^2^***: explained phenotypic variation.
