# Supplementary material for: Genetic dissection of grain water content and dehydration rate related to mechanical harvest in maize
Source: BMC Plant Biol. 2020 Mar 17;20:118. doi: 10.1186/s12870-020-2302-0 (PMC7076969; doi:10.1186/s12870-020-2302-0)
Supplement: Supplementary file 16 — Additional file 16: Table S10. Molecular markers developed in the QTL-qGwc1.1 region on chromosome 1. [file 12870_2020_2302_MOESM16_ESM.docx]

**Table S10** Molecular markers developed in the QTL-*qGwc1.1* region on chromosome 1

| **Marker** | **Forward primer** | **Reverse primer** | **Annealing temperature(℃)** | **Extension time(s)** | **Type** |
| --- | --- | --- | --- | --- | --- |
| SSR-62.1 | AAACAACAAGCGCAGAGAGG | GATGAGGCGAATCTAGGTGTC | 60 | 30 | SSR |
| SSR-63.1 | GATCCTGACCACGACGATGA | CTGCACCTCTTTGAGCCATC | 60 | 30 | SSR |
| SSR-63.2 | CGCTAGTCACAGCACCAATC | CCAAGCTACTGATGTCACCC | 60 | 30 | SSR |
| SSR-66.1 | AAAGCTCTCTCTTCCCCTGC | GGGGATGTTTTGTCAGCGAG | 60 | 30 | SSR |
| SSR-67.1 | TGATGGTGACCTTGGATCGT | ACCACTACCAGAACCACTCG | 60 | 30 | SSR |
| SSR-71.1 | ATCGCGATCCATTACCACCC | AGGCCTTACAGTACCCCAAC | 60 | 30 | SSR |
| SSR-73.1 | CGCCAGCCCTATTTTCACTG | AGTCTGGAGATGGAGCTTCG | 60 | 30 | SSR |
| SSR-75.1 | ACACGAGAGCTGGGTCAAAG | TTATGGACGAAGCCTGCGTA | 60 | 30 | SSR |
| SSR-78.1 | CCTCCACTTCCACGACTGTT | TCGTCTAGCAGCACCGAC | 60 | 30 | SSR |
| SSR-79.1 | TCTACTCATCATCTGGCCGG | GGAAGAAGGTCAGAGCTCGT | 60 | 30 | SSR |
| SSR-79.2 | CAATCATCGGGCGGGCTG | ACGGCAGAGTTACGTTCGAT | 60 | 30 | SSR |
| SSR-80.1 | CTTTCCTCAGCTTCGGGGTA | CAAGCGATTCCAAGAGCTCC | 60 | 30 | SSR |
| SSR-89.1 | TATCCTTAGTTTGTGCGCGC | GACAACGAGTAGTGGCAATCA | 55 | 30 | SSR |
| SSR-75.2 | TGTCACTGCCGAGCGATACTT | GCAAAACAGCACCCATCTGA | 60 | 30 | SSR |
| SSR-76.1 | CGAAAGGGCCGAAATCCAAA | TTCCTTGTCCTTCGTACCCC | 60 | 30 | SSR |
| SSR-77.1 | GAGATGCTCCTCCTCCTTCC | GAAGCTGGAGAGGGGAAGAG | 60 | 30 | SSR |
| SSR-79.3 | TGGGAAGAGTGGTAGTGGTG | TGTTGTTGTTGTTGCTGGCT | 60 | 30 | SSR |
| STS-76.1 | AGTGGAGGAAAATAGACCAGCT | AAACGTCGTCCAAACAGCTT | 60 | 30 | STS |
| STS-76.2 | GAATCGAACCAGGCGTCTTC | GCTTCTCCATGAGCGAGATC | 60 | 30 | STS |
| STS-78.2 | GGCCCATCCTATTGCTAAGG | GAAGATGGCCGCAACATAGG | 55 | 30 | STS |
| STS-78.3 | CCTGTTGCTTTATCATAAGGGGT | CAAAACGATCTAACTATGCCTCC | 55 | 30 | STS |
| STS-78.4 | CCGTCCGTCCAATGAATGTC | CGGAGTTGACGAGGGATCAA | 55 | 30 | STS |
| STS-79.4 | GCGGCATGTTACTCACCATG | GGTCATTGAGCGAGATTCAGT | 60 | 30 | STS |
| STS-77.2 | GGAATCATCTAAGAGCGCGG | GATGGCTGTGTGTGTCTTGG | 60 | 30 | STS |
| STS-78.5 | GGGATAATGCATGTGATTAGCGA | TCTCCGTTAAACAATGACCCA | 60 | 30 | STS |
| STS-78.6 | ACTGCGAAATGGGGTTTGAG | CAAGTTTCAGCTCGAGGACA | 60 | 30 | STS |
