# Supplementary material for: Genetic dissection of grain water content and dehydration rate related to mechanical harvest in maize
Source: BMC Plant Biol. 2020 Mar 17;20:118. doi: 10.1186/s12870-020-2302-0 (PMC7076969; doi:10.1186/s12870-020-2302-0)
Supplement: Supplementary file 17 — Additional file 17: Table S11. Comparison of the QTL detected in the current study with those revealed by other researchers. [file 12870_2020_2302_MOESM17_ESM.docx]

**Table S11** The comparison of consistent QTL with other researchers

| **QTL in our study** | **Consistent QTL in other research** | **References** |
| --- | --- | --- |
| *qGwc1.2* | Capelle et al.; Li et al. | [4, 43] |
| *qGwc2.3* | Capelle et al., Stuber et al., Melchinger et al. | [4, 61, 62] |
| *qGwc3.2* | Jia et al., Ho et al., Robertson et al. | [48, 63, 64] |
| *qGwc3.3* | Melchinger et al., Moreau et al. | [62, 65] |
| *qGwc4.1* | Stuber et al. | [61] |
| *qGwc4.2* | Li et al. | [43] |
| *qGwc5.2* | Stuber et al., Moreau et al. | [61, 65] |
| *qGwc7.3* | Capelle et al., Melchinger et al. | [4, 62] |
| *qGwc8.6* | Stuber et al. | [61] |
| *qGwc9.1* | Moreau et al. | [65] |
| *qGwc9.3* | Zhou et al. 2018 | [66] |
| *qGdr1.2* | Li et al., Dai et al. | [43, 47] |
| *qGdr3.3* | Wang et al., Dai et al. | [45, 47] |
| *qGdr5.1* | Liu et al., Wang et al. | [44, 45] |
| *qGdr8.3* | Wang et al., Dai et al. | [45, 47] |
